# Death Receptor (DR4) Haplotypes Are Associated with Increased Susceptibility of Gallbladder Carcinoma in North Indian Population

**DOI:** 10.1371/journal.pone.0090264

**Published:** 2014-02-28

**Authors:** Rajani Rai, Kiran L. Sharma, Surbhi Sharma, Sanjeev Misra, Ashok Kumar, Balraj Mittal

**Affiliations:** 1 Department of Genetics, Sanjay Gandhi Post Graduate Institute of Medical Sciences (SGPGIMS), Lucknow, India; 2 Department of Surgical Oncology, KGMU, Lucknow, India; 3 Department of Surgical Gastroenterology, Sanjay Gandhi Post Graduate Institute of Medical Sciences (SGPGIMS), Lucknow, India; National Cancer Center, Japan

## Abstract

**Background and Aim:**

Defective apoptosis is a hallmark of cancer development and progression. Death receptors (DR4, FAS) and their ligands (TRAIL, FASL) are thought to mediate the major extrinsic apoptotic pathway in the cell. SNPs in these genes may lead to defective apoptosis. Hence, the present study aimed to investigate the association of functional SNPs of DR4 (rs20575, rs20576 and rs6557634), FAS (rs2234767) and FASL (rs763110) with gallbladder cancer (GBC) risk.

**Methods:**

This case-control study included 400 GBC and 246 healthy controls (HC). Genotyping was carried out by Taqman genotyping assays. Statistical analysis was performed by using SPSS ver16. Meta-analysis was performed using Comprehensive Meta-analysis software (Version 2.0, BIOSTAT, Englewood, NJ) to systematically summarize the possible association of SNP with cancer risk. Functional prediction of these variants was carried out using Bioinformatics tools (FAST-SNP, F-SNP). False discovery rate (FDR test) was used in multiple comparisons.

**Results:**

The DR4 C_rs20575_A_rs20576_A_rs6557634_, G_rs20575_A_rs20576_G_rs6557634_ and G_rs20575_C_rs20576_G_rs6557634_ haplotypes conferred two-fold increased risk for GBC. Among these, the DR4 C_rs20575_A_rs20576_A_rs6557634_ haplotype emerged as main factor influencing GBC susceptibility as the risk was not modulated by gender or gallstone stratification. Our meta-analysis results showed significant association of DR4 rs6557634 with overall cancer risk, GI cancers as well as in Caucasians. We didn't find any association of FAS and FASL SNPs with GBC susceptibility.

**Conclusions:**

The DR4 haplotype C_rs20575_A_rs20576_A_rs6557634_ represents an important factor accounting the patients susceptibility to GBC probably due to decreased apoptosis. However, additional well-designed studies with larger sample size focusing on different ethnicities are required to further validate the results.

## Introduction

Gall bladder carcinoma (GBC) is the most common biliary tree cancer and fifth most common gastrointestinal malignancy, with striking geographical, age, race, and gender-related differences in its incidence [1]. Incidence of GBC in north India is 7.4 per 10^5^ for females and 3.6 per 10^5^ for males in Delhi [2]. The higher incidence of GBC in females has been attributed in part to hormonal factors, including estrogen and progesterone receptors. GBC is characterized by rapid progression and a very high mortality rate [3], [4]. Diagnosis of over 90% of GBC patients at an inoperable stage with serious invasion and metastasis to other organs is ascribed to absence of specific signs or symptoms [5]. Inadequate information and poor understanding regarding molecular basis of GBC [6] necessitate the establishment of important inroads in understanding the pathophysiology, early diagnosis and inter-individuals variability in susceptibility of GBC.

TRAIL-R1(TNF-related apoptosis-inducing ligand receptor 1)/DR4 and Fas/Apo-1/CD95 are members of tumor necrosis factor receptors which link exogenous stimuli via transmembrane surface receptors to the intracellular signaling machinery that mediates and executes the death signal. These pathways are one of the major extrinsic apoptotic signaling pathway [7] and defects in death receptor signaling can confer resistance to apoptosis. The role of apoptosis in tumorigenesis has been well-documented [8] and resistance to apoptosis is believed to be a hallmark of cancer [9]. Thus TRAIL/TRAIL-R and Fas/FasL system seems to play a crucial role in the pathogenesis of cancer. DR4 is thought to be an attractive candidate tumor suppressor gene [10]. DR4 and FAS mutations resulting loss or reduction of apoptotic function have been described in different human cancers [11], [12]. Aberrant expression of FAS and/or FASL has been detected in many human cancers and appears to be a feature of the malignant phenotype [13], [14].

Since single-nucleotide polymorphisms (SNPs) are the most common forms of human genetic variation and may affect cancer risk by influencing individual susceptibility [15], SNPs in DR4, FAS and FASL gene that impair the apoptotic signals represent the plausible and promising etiologic pathways modifying the GBC penetrance and merit to be tested as risk factors of gallbladder cancer susceptibility. The rs20575, located in exon 4, immediately 3′ to the main receptor ligand interface regions of the DR4 protein, results in the substitution of an arginine for threonine. It was suggested to influence the receptor ligand binding resulting deficient apoptotic signaling [11]. The rs20576 replaces glutamate by alanine which leads to structural change within region of TRAIL/DR4 complex formation. This may result in insufficient interaction with TRAIL and obstructed induction of caspase-8 dependent apoptosis, causing longer survival rate of tumor cells [16]. The FAS -1377 G to A substitution has been shown to decrease the expression of FAS protein [17] while the C allele of FASLG -844 C>T has been shown to increase the expression of the FASL protein [18]. Recently, many genetic studies have investigated the role of DR4, FAS and FASL polymorphisms in the etiology of various cancers [16], [19]–[30] etc. Nevertheless, the results of these studies remain inconclusive because some cancer types were in positively associated with these polymorphisms, while others were not. Therefore, we have carried out the present case-control study with the goal to find whether polymorphism of these apoptotic proteins modulates GBC susceptibility in North Indian population.

## Materials and Methods

### Ethics statement

The study protocol was sanctioned by the ethical committee of Sanjay Gandhi Post Graduate Institute of Medical Sciences (SGPGIMS) Lucknow (India) and King George Medical University (KGMU), Lucknow. The study subjects were registered according to the norms of the World's Association Declaration of Helsinki. Demographic characteristics data, such as gender, age, occupation history, tobacco history and other lifestyle factors, as well as clinical and pathological investigations were collected in a custom-designed questionnaire by conducting a personal interview with all study participants. Staging of cancer was done according to the AJCC-TNM classification system [31].

### Subjects

A total of 400 histo-pathologically confirmed gallbladder cancer patients from north Indian population were included in the present study. All the patients were enrolled from the Department of Gastroenterology of Sanjay Gandhi Postgraduate Institute of Medical Sciences, a tertiary care hospital in Lucknow, Uttar Pradesh, India and Department of Surgical Oncology, KGMU, Lucknow, Uttar Pradesh, India belonging to same ethnicity. For controls, 246 healthy individuals (age and sex matched with similar ethnicity to cases) unrelated to patients and to each other were recruited in the study from volunteers visiting the hospital for a routine checkup or health awareness camps and hospital employees from same region to ensure similar ethnicity. Samples were collected from June 2006 to Dec 2012.

The family history and general questionnaire of all the patients recruited for the study were taken to assure their ethnicity. The healthy controls had no evidence of any personal history of gallstone, gallbladder cancer or other malignant conditions or chronic disease. The inclusion and exclusion criteria for the recruitment of the Patients and controls were as previously described [32]. The participation rate was 100%, and blood samples were available for all subjects. 3 ml of blood was collected in EDTA vial and stored at −70°C. Informed and written consents were taken from all participants recruited in the study when interviewing for the demographic details and blood sample collection.

### DNA isolation

Genomic DNA was extracted from peripheral blood using the standard salting out method [33]. The quality and quantity of DNA were checked by using Nanodrop spectrophotometer (Thermo Fisher Scientific/Nanodrop Products, Wilmington, Delaware, USA).

### SNP selection

We have selected three coding and functional polymorphisms of DR4 gene (C>G/Thr209Arg/rs20575, A>C/Glu228Ala/rs20576 and G >A/Arg141His/rs6557634 which are the most commonly studied polymorphism. In addition, promoter polymorphism of FAS (-1377G/A, rs2234767) and FASL (– 844T/C, rs763110) believed to be involved in regulation of transcription were also included in the study.

### Genotyping of DR4, FAS and FASL polymorphisms

Taqman allelic discrimination assay on ABI 7500 Real Time PCR system using 96-well plates were used for genotyping of SNPs: rs20575, rs20576, rs2234767 and rs763110. Pre-designed assay primers and probes were purchased from Applied Biosystems (Foster City, CA, USA). A negative control (wells containing no DNA) was included in all plates. Amplification was detected by using ABI PRISM 7500 sequence detection software. SNP rs6557634 was analyzed using AS-PCR (Allele-specific Polymerase Chain Reaction). Details of the primers and cycle conditions for this SNP were taken from earlier published work [28]. Positive and negative controls were used in genotyping assay, and 5% of the samples were randomly selected and run in duplicates with 100% concordance. The results were consistent with no discrepancy in genotyping.

### Statistical analysis

Sample size was estimated via Quanto version 1.1.1 [34] using HapMap (http://hapmap.ncbi.nlm.nih.gov/) minor allele frequency data to attain 80% power. Hardy Weinberg Equilibrium in the control population was tested by employing Goodness of fit χ^2^ test.

All statistical analysis was performed using SPSS statistical analysis software, version 17.0 (SPSS, Chicago, IL, USA). Haplotypes estimation and linkage disequilibrium analysis was conducted using the SNPstat Software [35]. FAST-SNP (http://fastsnp.ibms.sinica.edu.tw) and F-SNP (http://compbio.cs.queensu.ca/F-SNP) was used for in-silico study.

Descriptive statistics of patients and controls were presented as the mean and standard deviations (SDs) for continuous measures, while frequencies and percentages were used for categorical measures. The differences in demographic variables and genotype distributions of the polymorphisms between cases and controls were compared by using Chi- square analysis or two-sided Fisher's exact test. The most common homozygous genotype was taken as a reference to calculate the risk estimates for codominant, dominant, and recessive genetic models. Binary logistic regression analysis was used for all analysis variables to estimate odds ratio (OR) and 95% confidence interval (CI) adjusted for age and gender to estimate the risk of gallbladder cancer with the polymorphisms. A two-tailed p-value of less than 0.05 was considered a statistically significant result. To keep meaningful results and maintain a low rate of false-positive findings, FDR was applied in case of multiple statistical testing [36].

### Meta-analysis

Literature was searched out in PubMed, Scopus and Embase, using the keywords: ‘DR4/rs20575/rs20576/rs6557634’, ‘polymorphism’, ‘cancer’. The search was updated extending all the published papers until July 2013. Reference lists of key studies and reviews were also hand-screened for additional potentially eligible studies. All the studies were included in the present meta-analysis if they met the following criteria: a) original papers, b). human-association studies, c). exploring the association between any of the three selected SNPs of DR4 gene and cancer susceptibility, d). case–control studies, e). papers with crude Odd's ratio and 95% confidence interval or sufficient data to calculate overall OR at 95% CI). Unpublished findings, reports without control population or genotype frequency data, duplication of previous publications and non-English articles were excluded. Information on the several data such as: first author's name, publication date, country, cancer type, ethnicity, number of patients/control included in the study, genotyping methods etc. was collected for each study. Ethnic groups were categorized as Asian, European and Mixed.

In meta-analysis, the strength of association between DR4 SNPs and risk of cancer was assessed by calculating the crude odds ratio (ORs) and corresponding 95% confidence interval (CI). Analyses were weighted by trial size. The pooled ORs were estimated for allele contrast (Variant vs. wild allele), heterozygote versus wild genotype, the homozygote variant genotype versus wild homozygote and also in dominant model. Statistical heterogeneity was evaluated using the Q statistic among the studies and it was considered as significant when p<0.05 [37]. Only random effects model (Der Simonian Laird) was used since it is more conservative. Publication bias was investigated with Begg's funnel plot. Funnel plot asymmetry was further assessed by the method of Egger's linear regression test with p<0.05 being considered statistically significant [38]. Moreover, sensitivity analysis was also performed, excluding studies whose allele frequencies in controls exhibited significant deviation from the Hardy–Weinberg equilibrium (HWE), given that the deviation may denote bias [39]. The significance of overall odds ratio (OR) was determined by the Z-test. All the p-values were two sided and all the statistical tests were performed using the Comprehensive Meta-analysis software (Version 2.0, BIOSTAT, Englewood, NJ).

## Results

### Population Characteristics

Table 1 presented the demographic characteristics of GBC patients with respect to their age and gender matched controls. The mean age of 400 GBC and 246 controls are 52.65±10.45 year and 47.75±10.65 year, respectively. There was no statistically significant difference among the mean age of controls and cases. About 95% of the GBC patients were in advanced stages of cancer (stage III and stage IV) and 31% of the GBC patients were tobacco users (either in some form smoking, chewing, or both). About 50.0% of GBC patients were associated with gallstones. All cancer patients were incident cases and none of the controls had a family history of cancer. Among GBC, 37% of the cases had early age of onset, i.e. <50 years.

**Table 1:** Demographic profile of the study subjects.

	GBC	HC
Variables	N (%)	N (%)
Subjects	400	246
Gender		
Male	122 (30.5)	83 (33.7)
Female	278 (69.5)	163 (66.3)
Age ±SD (year)	52.65±10.45	47.75±10.65
Gallstone		
No	200 (50.0 )	246 (100)
Yes	200 (50.0)	
Tobacco		
No	273 (68.9)	
Yes	123 (31.1)	
Stages		
0, I	No (0)	
II	21 (5.25)	
III	199 (49.75)	
IV	180 (45.0)	

HC - Healthy Control, GBC - Gallbladder cancer.

### Allelic distribution of studied polymorphisms in controls

The distributions of DR4, FAS and FASL polymorphisms (rs20575, rs20576, rs6557634, rs2234767 and rs763110) are shown in Table 2. The observed genotype frequencies of the two studied polymorphisms in HC were in accordance with Hardy-Weinberg equilibrium (p>0.05).

**Table 2:** Overall frequency distribution of DR4, FAS and FASL polymorphism in GBC and HC.

Genotype/Allele	HC N (%)	GBC N (%)	OR (95%CI)	p-value	FDR pcorr
DR4 rs20575 genotypes/alleles (age and gender adjusted)					
GG	128 (52.0)	183 (45.8)	Reference	-	-
CG	101 (41.1)	181 (45.2)	1.05 (0.69–1.59)	0.827	-
CC	17 (6.9)	36 (9.0)	1.94 (0.90–4.15)	0.090	-
P-trend				0.104	
CG+CC	118 (48.0)	217 (54.2)	1.16 (0.78–1.72)	0.472	-
GG+CG vs. CC	229 (93.1)	364 (91.0)	1.90 (0.91–3.97)	0.090	-
G	357 (72.6)	547 (68.4)	Reference	-	-
C	135 (27.4)	253 (31.6)	1.22 (0.90–1.67)	0.198	-
DR4 rs20576 genotypes/alleles (age and gender adjusted)					
AA	210 (85.4)	300 (75.0)	Reference	-	-
AC	33 (13.4)	86 (21.5)	1.74 (1.03–2.95)	0.039	0.043
CC	3 (1.2)	14 (3.5)	2.19 (0.47–10.19)	0.320	-
P-trend				0.001	
AC+CC	36 (14.6)	100 (25.0)	1.78 (1.07–2.95)	0.026	0.035
AA+AC vs. CC	243 (98.8)	386 (96.5)	1.97 (0.42–9.17)	0.390	-
A	453 (92.1)	686 (85.8)	Reference	-	-
C	39 (7.9)	114 (14.2)	1.71 (1.08–2.71)	0.023	0.033
DR4 rs6557634 genotypes/alleles (age and gender adjusted)					
GG	126 (51.2)	174 (43.5)	Reference	-	-
GA	107 (43.5)	192 (48.0)	1.61 (1.06–2.44)	0.025	0.035
AA	13 (5.3)	34 (8.5)	2.05 (0.90–4.70)	0.088	-
P-trend				0.029	
GA+AA	120 (48.8)	226 (56.5)	1.66 (1.11–2.48)	0.013	0.028
GG+GA vs. AA	233 (94.7)	366 (91.5)	1.63 (0.73–3.62)	0.234	-
G	359 (73.0)	540 (67.5)	Reference	-	-
A	133 (27.0)	260 (32.5)	1.45 (1.07–1.98)	0.018	0.030
FASL rs763110 genotypes/alleles (age and gender adjusted)					
TT	90 (36.6)	145 (36.2)	Reference	-	-
CT	119 (48.4)	180 (45.0)	0.97 (0.63–1.50)	0.898	-
CC	37 (15.0)	75 (18.8)	1.14 (0.64–2.03)	0.667	-
P-trend				0.048	
CT+TT	156 (63.4)	255 (63.8)	1.01 (0.67–1.53)	0.947	-
TT+CT vs. CC	209 (85.0)	325 (81.2)	1.15 (0.68–1.96)	0.598	-
T	299 (60.8)	470 (58.8)	Reference	-	-
C	193 (39.2)	330 (41.2)	1.05 (0.79–1.40)	0.739	-
FAS rs2234767 genotypes/alleles (age and gender adjusted)					
GG	153 (62.2)	245 (61.2)	Reference	-	-
AG	86 (35.0)	136 (34.0)	0.95 (0.62–1.44)	0.802	-
AA	7 (2.8)	19 (4.8)	1.68 (0.56–5.07)	0.356	-
P-trend				0.537	
AG+AA	93 (37.8)	155 (38.8)	1.00 (0.67–1.50)	0.997	-
GG+AG vs. AA	239 (97.2)	381 (95.2)	1.72 (0.56–5.11)	0.333	-
G	392 (79.7)	626 (78.2)	Reference	-	-
A	100 (20.3)	174 (21.8)	1.06 (0.75–1.49)	0.753	-

HC - Healthy Control, GBC - Gallbladder cancer, OR - Odds Ratio, CI - Confidence Interval.

Significant Values are given in bold.

### Association of DR4, FAS and FASL polymorphisms with GBC

The risk of GBC in relation to each of the SNPs studied in DR4, FAS and FASL genes are shown in Table 2. On comparing the genotype frequency distribution of DR4 SNPs (rs20575, rs20576and rs763110) in GBC patients with that of controls, we found a significant association of rs20576 with GBC risk at heterogenotype (AC, pcorr = 0.043, OR = 1.74), variant allele (C, pcorr = 0.033, OR = 1.71) as well as in dominant model (AC+CC, pcorr = 0.035, OR = 1.78). Similarly, heterozygous carriers of DR4 SNP rs6557634 also showed a significant association with increased gallbladder cancer risk (GA, pcorr = 0.035, OR = 1.61). This association also persisted at allele level (A, pcorr = 0.030, OR = 1.45) as well as in dominant model (GA+AA, pcorr = 0.028, OR = 1.66). In contrast, there were no significant differences in genotype frequencies between gallbladder cancer cases and controls for DR4 rs20575.

In haplotype analysis, the DR4 haplotype comprising the major alleles was taken as reference and the difference in the frequencies of haplotypes between patients and controls were tested using chi-square test (Table 3). The haplotype analyses of the three studied SNPs of DR4 revealed that the frequencies of C_rs20575_A_rs20576_A_rs6557634_, G_rs20575_A_rs20576_G_rs6557634_ and G_rs20575_C_rs20576_G_rs6557634_ haplotypes were significantly higher in GBC as compared to controls (pcorr = <0.0005, OR = 2.76; pcorr =  0.0244, OR = 2.09 and pcorr = 0.0335, OR = 2.80, respectively). These haplotypes were found to confer two-fold increased risk for GBC. The global haplotype analysis also demonstrated statistically significant differences (pcorr = 0.0015) between GBC cases and controls based on the distribution pattern of the all haplotypes (Table 3).

**Table 3:** Frequency distribution of DR4 haplotypes in GBC patients and HC.

Haplotypes	Frequency		p- value	OR (95%CI)
	HC 246 (%)	GBC 400 (%)		
C_rs20575_A_rs20576_G_rs6557634_	0.5917	0.4316	---	Reference
C_rs20575_A_rs20576_A_rs6557634_	0.119	0.2095	<0.0001^a^	2.76 (1.71 – 4.47)
G_rs20575_A_rs20576_G_rs6557634_	0.0947	0.1427	0.0084^b^	2.09 (1.21 – 3.62)
G_rs20575_A_rs20576_A_rs6557634_	0.1154	0.0737	0.62	0.86 (0.47 – 1.57)
G_rs20575_C_rs20576_G_rs6557634_	0.0306	0.0719	0.022^c^	2.80 (1.16–6.76)
G_rs20575_C_rs20576_A_rs6557634_	0.0337	0.028	0.23	1.75 (0.71–4.32)
C_rs20575_C_rs20576_G_rs6557634_	0.0127	0.0288	0.31	1.83 (0.57–5.92)
Global haplotype association p-value: 0.00038^d^				

HC - Healthy control, GBC - Gallbladder cancer, OR - Odds Ratio, CI - Confidence Interval.

Significant Values are given in bold, FDR pcorr = 0.0005**^a^**, 0.0244**^b^**, 0.0335**^c^**, 0.0015**^d^**.

However, no significant differences were observed for, FAS rs2234767 and FASLrs763110 SNP in any groups both at genotypic and allelic levels as well as for dominant and recessive models (Table 2).

### Association of DR4, FAS and FASL polymorphisms with GBC after Gender stratification

Stratification of subjects on the basis of gender showed that the C_rs20575_A_rs20576_A_rs6557634_ and G_rs20575_A_rs20576_G_rs6557634_ haplotypes of DR4 were significantly associated with a two-fold increased risk of GBC in females (pcorr = 0, OR = 2.60 and pcorr =  0.043, OR = 2.10, respectively]. However, the C_rs20575_A_rs20576_A_rs6557634_ haplotype was found to confer increased risk of GBC in males (pcorr = 0.0277, OR = 3.55) as compared to controls (Table 4). The global haplotype association was also significant only in female GBC patients (pcorr = 0.0245) as compared to controls.

**Table 4:** Haplotype Analysis of DR4 gene in GBC and HC after gender stratification.

Haplotypes	Haplotype Analysis of DR4 in male				Haplotype Analysis of DR4 in females			
	Frequency				Frequency			
	HC (83)	GBC (122)	p- value	OR (95%CI)	HC (163)	GBC (278)	p- value	OR (95%CI)
C_rs20575_A_rs20576_G_rs6557634_	0.5826	0.4181	---	Reference	0.6008	0.4366	---	Reference
C_rs20575_A_rs20576_A_rs6557634_	0.1309	0.2351	0.013^a^	3.55 (1.32–9.54)	0.1079	0.1984	0.0000^b^	2.60 (1.49 –4.52)
G_rs20575_A_rs20576_G_rs6557634_	0.1122	0.1435	0.069	2.44 (0.94–6.34)	0.084	0.1435	0.035^c^	2.10 (1.06 – 4.17)
G_rs20575_A_rs20576_A_rs6557634_	0.108	0.0681	0.74	0.81 (0.22–2.90)	0.1215	0.0758	0.066	0.86 (0.43–1.70)
G_rs20575_C_rs20576_G_rs6557634_	0.016	0.0447	0.092	9.10 (0.71–117.02)	0.0392	0.0803	0.073	2.31 (0.93–5.78)
G_rs20575_C_rs20576_A_rs6557634_	0.0349	0.0265	0.34	0.57(0.07–4.89)	0.0314	0.0313	0.12	2.39 (0.80–7.19)
C_rs20575_C_rs20576_G_rs6557634_	0	0.0495	0.61	5.57 (0.17–186.44)	0.0153	0.0231	0.59	1.43 (0.38–5.36)
C_rs20575_C_rs20576_A_rs6557634_	0.0154	0.0145	0.36	4.78 (0.17–136.99)	NA	0.011	0.0000	……
	Global haplotype association p-value = 0.11				Global haplotype association p-value = 0.0092^d^			

GBC - Gallbladder cancer, HC - Healthy controls, OR - Odds Ratio, CI - Confidence Interval

Significant Values are given in bold, FDR pcorr = 0.0277 ^a^, 0 ^b^, 0.0430 ^c^, 0.0245^d^.

No association was observed between FASL SNP rs763110 and GBC susceptibility in both males and females. However, the variant allele of FAS SNP rs2234767 was found to confer significantly protective effect for GBC in males (A; pcorr = 0.043, OR = 0.51, Table S1 in File S1).

### Association of DR4, FAS and FASL polymorphisms with GBC after gallstone stratification

Since GBC is often associated with gallstones, we stratified patients on the basis of presence/absence of gallstone to see the combined effect of gallstone and DR4, FAS and FASL variants on GBC risk. A significant difference was found for DR4 C_rs20575_A_rs20576_A_rs6557634_, and G_rs20575_C_rs20576_A_rs6557634_ haplotypes in GBC with stones as compared to controls (pcorr = <0.0005, OR = 4.04 and pcorr = 0.0277, OR = 3.78, respectively). Similarly, C_rs20575_A_rs20576_A_rs6557634_, G_rs20575_A_rs20576_G_rs6557634_ and G_rs20575_C_rs20576_G_rs6557634_ haplotypes of DR4 gene were found to significantly higher in GBC without gallstone as compared with control (pcorr = 0.0282, OR = 1.97; pcorr = 0.0154, OR = 2.35 and pcorr = 0.0434, OR = 2.81, respectively). The global haplotype association in GBC patients with and without stone was also significant (pcorr = <0.0005 and 0.0277, respectively; Table 5).

**Table 5:** Haplotype analysis of DR4 gene in GBC (with stone and without stone) and HC.

	Haplotype Analysis of DR4 in GBC without stone and HC				Haplotype Analysis of DR4 in GBC with stone and HC			
	Frequency				Frequency			
Haplotypes	HC (246)	GBC (200)	p- value	OR (95%CI)	HC (246)	GBC (200)	p- value	OR (95%CI)
C_rs20575_A_rs20576_G_rs6557634_	0.5917	0.4271	---	Reference	0.5917	0.4399	---	Reference
C_rs20575_A_rs20576_A_rs6557634_	0.119	0.1978	0.015^ a^	1.97 (1.14 -3.41)	0.119	0.2165	<0.0001^e^	4.04 (2.23 –7.32)
G_rs20575_A_rs20576_G_rs6557634_	0.0947	0.1666	0.0048^ b^	2.35 (1.30–4.23)	0.0947	0.115	0.24	1.58 (0.74– 3.35)
G_rs20575_A_rs20576_A_rs6557634_	0.1154	0.0659	0.32	0.69 (0.34 – 1.42)	0.1154	0.086	0.86	1.07 (0.50 – 2.30)
G_rs20575_C_rs20576_G_rs6557634_	0.0306	0.0737	0.042^ c^	2.81 (1.04–7.61)	0.0306	0.0695	0.075	2.61 (0.91–7.46)
G_rs20575_C_rs20576_A_rs6557634_	0.0337	0.0187	0.94	0.95 (0.27–3.33)	0.0337	0.0369	0.013^f^	3.78 (1.32–10.79)
C_rs20575_C_rs20576_G_rs6557634_	0.0127	0.0375	0.21	2.26 (0.64–7.99)	0.0127	0.0205	0.52	1.60 (0.38–6.67)
	Global haplotype association p-value: 0.016^d^				Global haplotype association p-value: <0.0001^g^			

GBC-Gallbladder cancer, HC-Healthy controls, OR -Odds Ratio, CI-Confidence Interval

Significant Values are given in bold, FDR pcorr = 0.0282^a^, 0.0154^b^, 0.0434^c^, 0.0284^d^, 0.0005^e^, 0.0277^f^, 0.0005^g^.

Table S2 in File S1 shows the distribution of SNPs after stratifying the subjects on the basis of gallstone. The frequencies of FAS and FASL genotypes did not differ significantly between control and GBC with or without stones, showing lack of association (Table S2 in File S1).

### Meta-analysis for DR4 rs20575

There were fourteen studies investigating the association of DR4rs20575 with cancer risk [10], [19], [21], [22], [25], [28], [40]–[46] but we have excluded two studies of Kazuya Kuraoka 2005 [43] and Verònica Fernàndez 2004 [44] because these studies either deviated from HWE (p<0.05) [43] or used tissue samples [44] for polymorphism analysis. We combined the results of our study with the rest 12 eligible studies [10], [19], [21], [22], [25], [28], [40]–[42], [45], [46] to calculate pooled OR for 4731 cases/5029 control and found no significant overall association with risk of cancer [C vs. G: OR = 1.05 (0.97–1.12), p = 0.222; CC vs. GG: OR = 1.07 (0.94–1.22), p = 0.291; CG vs. GG: OR = 1.08 (0.92–1.27), 0.352; CC+CG vs. GG: OR = 1.09 (0.94–1.26), p = 0.268; Table 6 and Figure S1 in File S1]. On subgroup analysis, no significant association was found amongst any groups after ethnicity or cancer site based subgroupings.

**Table 6:** Meta-analysis Results.

DR4 rs20575																		
Variables	N		Case/Control	C vs G allele			CC vs GG				CG vs GG			CC+CG vs GG				
				OR (95% CI)	p	P_h_/I^2^	OR (95% CI)	p	P_h_/I^2^		OR (95% CI)	p	P_h_/I^2^	OR (95% CI)	p	P_h_/I^2^		
All	13		4731/5029	1.05 (0.97–1.12)	0.222	0.203/23.764	1.07 (0.94–1.22)	0.291	0.363/8.328		1.08 (0.92–1.27)	0.352	0.830/0.000	1.09 (0.94–1.26)	0.268	0.011/53.862		
Ethnicity																		
Caucassian	9		4304/4578	1.06 (0.98–1.16)	0.153	0.095/40.807	1.11 (0.95–1.30)	0.192	0.200/27.484		1.07 (0.89–1.29)	0.446	0.002/67.245	1.10 (0.93–1.30)	0.282	0.004/64.711		
Mixed	3		292/282	1.04 (0.82–1.31)	0.764	0.594/0.000	1.08 (0.67–1.72)	0.764	0.586/0.000		1.31 (0.82–2.10)	0.258	0.295/18.173	1.22 (0.82–1.81)	0.323	0.418/0.000		
Asian	1		135/169	0.87 (0.63–1.20)	0.380	1.000/0.000	0.79 (0.43–1.44)	0.435	1.000/0.000		0.84 (0.50–1.41)	0.511	1.000/0.000	0.82 (0.51–1.32)	0.413	1.000/0.000		
Cancer type																		
GI Cancer	6		1862/1821	1.12 (0.98–1.28)	0.086	0.116/43.318	1.26 (0.98–1.62)	0.071	0.237/26.340		1.15 (0.85–1.56)	0.354	0.002/73.820	1.18 (0.90–1.54)	0.235	0.004/70.920		
Other Cancer	7		2869/3208	0.99 (0.92–1.06)	0.728	0.796/0.000	0.98 (0.85–1.14)	0.786	0.804/0.000		0.98 (0.84–1.14)	0.761	0.250/23.434	0.96 (0.86–1.08)	0.516	0.436/0.000		
DR4 rs20576																		
Variables	N		Case/Control	C vs A allele			CC vs AA				AC vs AA			CC+AC vs AA				
				OR (95% CI)	p	P_h_/I^2^	OR (95% CI)	p	P_h_/I^2^		OR (95% CI)	p	P_h_/I^2^	OR (95% CI)	p		P_h_/I^2^	
All	11		2589/3222	1.23 (0.95–1.59)	0.112	0.000/81.444	1.48 (0.85–2.57)	0.162	0.014/54.807		1.16 (0.87–1.53)	0.310	0.000/76.133	1.21 (0.91–1.62)	0.192		0.000/79.793	
Ethnicity																		
Caucassian	8		2297/2940	1.32 (0.97–1.80)	0.076	0.000/86.343	1.60 (0.87–2.95)	0.131	0.007/63.824		1.19 (0.85–1.67)	0.300	0.000/81.886	1.28 (0.91–1.82)	0.162		0.000/85.004	
Mixed	3		292/282	0.98 (0.69–1.41)	0.930	0.342/6.577	0.98 (0.20–4.75)	0.977	0.255/26.719		1.02 (0.61–1.71)	0.931	0.213/35.309	1.00 (0.64–1.55)	0.994		0.289/19.400	
Cancer type																		
GI Cancer	5		1653/1574	1.24 (0.84–1.84)	0.285	0.000/87.721	1.43 (0.70–2.91)	0.327	0.030/62.643		1.18 (0.79–1.76)	0.430	0.000/81.897	1.23 (0.79–1.92)	0.358		0.000/86.120	
Other Cancer	6		936/1648	1.23 (0.82–1.86)	0.319	0.001/75.590	1.91 (0.57–6.41)	0.295	0.049/54.975		1.14 (0.70–1.84)	0.608	0.002/73.815	1.20 (0.76–1.91)	0.436		0.001/74.698	
DR4 rs6557634																		
Variables	N	Case/Control		A vs G allele			AA vs GG			AG vs GG				AA+AG vs GG				
				OR (95% CI)	p	P_h_/I^2^	OR (95% CI)	p	P_h_/I^2^	OR (95% CI)		p	P_h_/I^2^	OR (95% CI)	p			P_h_/I^2^
All	4	832/723		1.22(1.05–1.42)	0.011	0.604/0.000	1.52(1.06–2.19)	0.023	0.390/0.367	1.24 (0.99–1.54)		0.059	0.830/0.000	1.29 (1.05–1.60)	0.017			0.909/0.000
Ethnicity																		
Caucassian	2	600/471		1.31 (1.08–1.58)	0.005	0.923/0.000	2.03 (1.23–3.34)	0.006	0.771/0.000	1.26 (0.97–1.62)		0.079	0.748/0.000	1.34 (1.05–1.71)	0.019			0.875/0.000
Mixed	2	232/252		1.07 (0.83–1.37)	0.623	0.658/0.000	1.11 (0.66–1.88)	0.692	0.588/0.000	1.18 (0.76–1.83)		0.464	0.397/0.000	1.16 (0.77–1.77)	0.480			0.668/0.000
Cancer type																		
GI Cancer	2	600/471		1.31 (1.08–1.58)	0.005	0.923/0.000	2.03 (1.23–3.34)	0.006	0.771/0.000	1.26 (0.97–1.62)		0.079	0.748/0.000	1.34 (1.05–1.71)	0.019			0.875/0.000
Other Cancer	2	232/252		1.07 (0.83–1.37)	0.623	0.658/0.000	1.11 (0.66–1.88)	0.692	0.588/0.000	1.18 (0.76–1.83)		0.464	0.397/0.000	1.16 (0.77–1.77)	0.480			0.668/0.000

Significant associations are shown in bold, Ph- p-value of Q test for heterogeneity, OR -Odds Ratio, CI-Confidence Interval.

### Meta-analysis for DR4 rs20576

For rs20576, 8 studies were found eligible [10], [16], [19], [22], [28], [40]–[42]. Wolf et al., 2006 [16] conducted the association in CLL, MCL, bladder cancer, prostate cancer and HNSCC. These studies were taken as individual study because of different type of cancer; however we have excluded the studies involving HNSCC and prostate cancer as tissue samples were used in analysis. Thus, a total of 11 studies (Table S3 in File S1) including our study, having a total of 2589 cases/3222 controls (400 patients/246 controls from present study) were included. The meta-analysis results indicated heterogeneity, hence random model was used for meta-analysis [phet = <0.05, Figure S2 in File S1]. Overall, this SNP did not show any association with cancer risk [C vs. A: OR = 1.23 (0.95–1.56), p = 0.112; CC vs. AA: OR = 1.48 (0.85–2.57), p = 0.162; AC vs. AA: OR = 1.16 (0.87–1.53), p = 0.310; CC+AC vs. AA = 1.21 (0.91–1.62), p = 0.192]. In subgroup analysis, also, rs20576 did not show any significant association with cancer risk.

### Meta-analysis for DR4 rs6557634

For rs6557634, only three studies were found [19], [28], [42] and on combining the results of these studies with our study (total 832 patients/723 control), overall a marginal significant association was found for cancer risk with A vs. G [OR = 1.22 (1.05–1.42), p = 0.011], AA vs. GG [OR = 1.52 (1.06–2.19), p = 0.023] and in dominant model [AA+AG vs. GG: OR = 1.29 (1.05–1.60), p = 0.017, Figure S3 in File S1]. In subgroup analysis these association were found limited to only Caucasians and for GI cancer (Table 6).

### Bias diagnostics

Funnel plot shape for the pooled analysis did not reveal any asymmetry in all comparison models. Egger's test also did not suggest any evidence of publication bias for rs20575 [CC vs. GG: t = 1.28, p = 0.228; CG vs. GG: t = 2.12, p = 0.057; CG+CC vs. GG: t = 1.89, p = 0.086; and C vs. G allele: t = 1.13, p = 0.281, Figure S4 in File S1], for rs20576 [CC vs. AA: t = 1.49, p = 0.170; AC vs. AA: t = 0.66, p = 0.525; AC+CC vs. AA: t = 0.90, p = 0.392; C vs. A allele: t = 1.08, p = 0.308, Figure S5 in File S1] and for rs6557634 [AA vs. GG: t = 0.91, p = 0.460; AG vs. GG: t = 0.47, p = 0.687; AG+AA vs. GG: t = 1.87, p = 0.202; A vs. G allele: t = 2.82; p = 0.106, Figure S6 in File S1].

### *In-silico* analysis

Table S4 in File S1 show our in-silico analysis for the five studied SNPs. The rs20575 was found to be involved in splicing regulation, though the effect was tolerated and benign (FS score = 0.330). Similarly, rs6557634 and was found to be involved in splicing, possibly damaging and benign (FS score = 0.284). The rs20576 was also found to be possible damaging but tolerated. The rs763110 was involved in transcriptional regulation while rs2234767 was found benign.

## Discussion

TRAIL-R1 and Fas death receptors form a subgroup of the tumor necrosis factor receptors (TNF-R) superfamily having a conserved cytoplasmic signaling module (≈80 amino acid) termed the death domain DD which is essential for apoptosis induction [47]. Binding of these receptors to their cognate ligands (i.e., TRAIL and FASL, respectively) lead to receptor aggregation and recruitment of adaptor proteins, and caspases, forming the Death-Inducing Signaling Complex (DISC). This, in turn, activate and releases caspase 8 and 10 for the triggering of apoptosis [48]. Normal variations within the sequence of apoptotic genes are suggested to lead suboptimal apoptotic capacity, finally increasing cancer risk [49]. Despite many investigations, the associations between DR4, FAS, and FASL polymorphisms and the risk of human cancers remain inconsistent between different studies. Therefore, in this study, we tested the association of coding polymorphisms of the death receptor 4 (DR4), FAS, and its legend (FASL) with GBC susceptibility. In addition a meta-analysis was also performed to find out the overall cancer risk.

The rs20575, was suggested to result deficient apoptotic signaling [11]. However, we did not find any association of DR4 rs20575 polymorphism with the GBC risk. Our results are in agreement with other studies showing null association of rs20575 with lung cancer [28], breast cancer [10], [25], [42], [45], bladder cancer [19], and gastric cancer [43], [50]. In our meta-analysis also, we didn't find any association with cancer risk. A previous meta-analysis by Chen et al., 2009 also indicated only marginal association of rs20575 with overall cancer susceptibility [51]. Our in-silico polyphen analysis has also showed rs20575 to be benign.

For DR4 rs20576, we have observed an increased risk of GBC at hetero-genotype level, allelic level and in dominant model. Our in-silico polyphen study also showed rs20576 to be possibly damaging. The rs20576 variant was found to exhibit an enhanced risk to have CLL, MCL, HNSCC and bladder cancer as well as an enhanced risk for men to have prostate cancer [16]. Additionally, rs20576 variant carriers exhibited significantly enhanced colorectal cancer risk dependent on allele dose for female and also with advanced colorectal cancer stages [22]. However, some studies have also reported null association of this SNP with bladder [19], breast [10], [42] and lung cancers [28], [40]. In meta-analysis study, Chen et al also indicated increased risk of all types of cancer with AC and CC variants of rs20576 [51], but we failed to show any association with overall cancer risk and subgroups.

We found the association of DR4 rs6557634 with GBC risk. Previously, Mittal RD et al., 2011 [19], also reported significant association of variant genotype of this SNP with bladder cancer risk. Though, Ulybina et al., 2009 [28] and Ulybina et al., 2011 [42] failed to find any association with lung and breast cancer respectively. Our meta-analysis results showed a significant association of rs6557634 with overall cancer risk, GI cancers as well as in Caucasians. In-silico study using polyphen also showed this SNP to be possibly damaging.

In our study, three haplotypes of DR4 C_rs20575_A_rs20576_A_rs6557634_, G_rs20575_A_rs20576_G_rs6557634_ and G_rs20575_C_rs20576_G_rs6557634_ were associated with increased risk of GBC. The C_rs20575_A_rs20576_A_rs6557634_ haplotype persisted as risk factor even after gender or gallstone stratification of GBC cases. The C_rs20575_-C_rs20576_ diplotype was previously found to confer 2.4-fold risk of colorectal cancer by Frank et al., 2006 [22] and 3.5-fold risk of breast cancer by Frank et al., 2005 [10]. In another study, C_rs20575_-G_rs6557634_-C_rs20576_ haplotype carriers had 1.8 folds increased risk in bladder cancer patients [19]. Thus, haplotypes seems to be better in predicting the association of DR4 with GBC risk rather than individual SNPs.

FAS is a cell surface receptor found expressed in a variety of tissues and FASL is the natural ligand to FAS, whose expression is restricted to the activated T cells and natural killer cells [52]. The decreased expression of FAS may keep the transformed cells from elimination by antitumor immune response, called immune escape while the increased expression of FASL may increase the ability of tumor cells to kill FAS-sensitive lymphocytes, called immune counterattack [53]. Recently, up-regulation of FasL expression was reported in gallbladder carcinoma cells suggesting that FASL plays an important role in invasive depth, histological classification and metastasis of gallbladder carcinoma [54]. However, we failed to find any association of FAS (1377 G>A) and FASL (-844 C>T) polymorphisms with GBC susceptibility. Since these are receptor ligand system, an apoptotic cell death needs both normal FAS and normal FASL [14]. We have also looked for genotypic interactions between these two SNPs but we failed to find such an association (data not shown). Similarly, several other investigators have also failed to find an association of these SNP with cervical cancer [55], AML [56], lung cancer [57], gastric cancer [58] and NSCLC risk [27]. A meta-analysis by Zhang et al., 2009 [59] suggested that the FAS -1377G to A polymorphism may be a low-penetrance susceptibility marker of cancer. Another meta-analysis study by Zhang et al., 2009 [53] suggested a possible protective effect of FASL rs763110 T allele on cancer risk. These findings suggest that the promoter polymorphisms of the FAS and FASLG genes may not contribute to the etiology of cancer [58] and mutation of the primary structure of FAS or FASL might be one of the possible mechanisms that disrupt FAS-mediated apoptosis in tumor cells.

FASL expression has been found to be induced by tobacco smoking [60]. DNA damage resulting from procarcinogenic compounds contained in tobacco smoke may increase DR4 transcription and apoptotic cascade [61]. Considering the plausible combined effect of SNP and environmental exposure of various carcinogens like tobacco/cigarette to modify individual's susceptibility to cancer, we have also looked for this in case only study. However, we did not found any association of the combined effect of tobacco and these SNPs in modulating the GBC susceptibility (data not shown).

To the best of our knowledge, this is the first study investigate the effect of coding SNPs of DR4, FAS and FASL in predicting the GBC risk. The different effects of these aforesaid SNP on the various cancer risks may be partially ascribed to different molecular mechanisms underlying the pathogenesis of different cancers. Even in the same disease, different results may be due to difference in the genetic background in different ethnicities and/or influence of different environmental factors. The allelic and genotype frequencies of these SNPs vary greatly in different ethnic groups. In addition, inadequate study design such as nonrandom sampling, limited sample size and the pitfalls arising from unknown confounders also need be considered.

## Conclusion

DR4 haplotypes, especially C_rs20575_A_rs20576_A_rs6557634_, significantly increased the GBC risk and was unaffected by gender and gallstone status of patients. This haplotype may change the apoptotic signals, thus may modulate cancer susceptibility probably by promoting tumor cells survival and tumor growth rather than initiating tumor formation. It represents a late event and useful biomarker for GBC susceptibility. Though, it needs to re-confirmed in larger population based cohorts and validate in GBC patients of different ethnicities. In contrast, polymorphism of FAS and FASL system might not have any effect on influencing GBC susceptibility in North Indian population. Since a better understanding of the regulation of the signaling events and their perturbation in human cancers may lead to the identification of new molecular targets that can be exploited for therapeutic purposes. This strategy is expected to open new perspectives to target the death receptor pathway for cancer therapy.

## Supporting Information

File S1 **Figure S1.** Forest plot for rs20575. **Figure S2.** Forest plot for rs205756. **Figure S3.** Forest plot for rs6557634. **Figure S4.** Funnel plot for rs20575. Each dot represents an individual study for the indicated association. The horizontal lines represent CIs. **Figure S5.** Funnel plot for rs205756. Each dot represents an individual study for the indicated association. The horizontal lines represent CIs. **Figure S6.** Funnel plot for rs6557634. Each dot represents an individual study for the indicated association. The horizontal lines represent CIs. **Table S1.** Frequency distribution of DR4, FAS and FASL Gene polymorphism in GBC and HC after subdividing on the basis of gender. **Table S2**. Frequency distribution of polymorphism in GBC and HC after subdividing on the basis of gallstone status. **Table S3.** Studies included in meta-analysis. **Table S4:** Functional information
(ZIP)

## References

[1] Hamdani NH, Qadri SK, Aggarwalla R, Bhartia VK, Chaudhuri S, et al (2012) Clinicopathological study of gall bladder carcinoma with special reference to gallstones: our 8-year experience from eastern India. Asian Pac J Cancer Prev 13: 5613–5617. 23317226 10.7314/apjcp.2012.13.11.5613

[2] Murthy NS, Rajaram D, Gautam M (2011) Trends in incidence of gallbladder cancer–Indian scenario. Gastrointestinal cancer: Targets and therapy 1: 1–9.

[3] Jin K, Lan H, Zhu T, He K, Teng L (2011) Gallbladder carcinoma incidentally encountered during laparoscopic cholecystectomy: how to deal with it. Clin Transl Oncol 13: 25–33. 21239352 10.1007/s12094-011-0613-1

[4] Kalita D, Pant L, Singh S, Jain G, Kudesia M, et al (2013) Impact of routine histopathological examination of gall bladder specimens on early detection of malignancy - a study of 4,115 cholecystectomy specimens. Asian Pac J Cancer Prev 14: 3315–3318. 23803122 10.7314/apjcp.2013.14.5.3315

[5] Miao X, Yang ZL, Xiong L, Zou Q, Yuan Y, et al (2013) Nectin-2 and DDX3 are biomarkers for metastasis and poor prognosis of squamous cell/adenosquamous carcinomas and adenocarcinoma of gallbladder. Int J Clin Exp Pathol 6: 179–190. 23330003 PMC3544223

[6] Ghosh M, Sakhuja P, Singh S, Agarwal AK (2013) p53 and beta-catenin expression in gallbladder tissues and correlation with tumor progression in gallbladder cancer. Saudi J Gastroenterol 19: 34–39. 23319036 10.4103/1319-3767.105922 PMC3603488

[7] Guicciardi ME, Gores GJ (2009) Life and death by death receptors. FASEB J 23: 1625–1637. 19141537 10.1096/fj.08-111005 PMC2698650

[8] Zornig M, Hueber A, Baum W, Evan G (2001) Apoptosis regulators and their role in tumorigenesis. Biochim Biophys Acta 1551: F1–37. 11591448 10.1016/s0304-419x(01)00031-2

[9] Fulda S (2009) Tumor resistance to apoptosis. Int J Cancer 124: 511–515. 19003982 10.1002/ijc.24064

[10] Frank B, Hemminki K, Shanmugam KS, Meindl A, Klaes R, et al (2005) Association of death receptor 4 haplotype 626C-683C with an increased breast cancer risk. Carcinogenesis 26: 1975–1977. 15975957 10.1093/carcin/bgi164

[11] Fisher MJ, Virmani AK, Wu L, Aplenc R, Harper JC, et al (2001) Nucleotide substitution in the ectodomain of trail receptor DR4 is associated with lung cancer and head and neck cancer. Clin Cancer Res 7: 1688–1697. 11410508

[12] Lee SH, Shin MS, Park WS, Kim SY, Dong SM, et al (1999) Alterations of Fas (APO-1/CD95) gene in transitional cell carcinomas of urinary bladder. Cancer Res 59: 3068–3072. 10397246

[13] Kang S, Dong SM, Seo SS, Kim JW, Park SY (2008) FAS -1377 G/A polymorphism and the risk of lymph node metastasis in cervical cancer. Cancer Genet Cytogenet 180: 1–5. 18068525 10.1016/j.cancergencyto.2007.09.002

[14] Zhang X, Miao X, Sun T, Tan W, Qu S, et al (2005) Functional polymorphisms in cell death pathway genes FAS and FASL contribute to risk of lung cancer. J Med Genet 42: 479–484. 15937082 10.1136/jmg.2004.030106 PMC1736067

[15] Zhang H, Ma H, Li L, Zhang Z, Xu Y (2013) Association of methylenetetrahydrofolate dehydrogenase 1 polymorphisms with cancer: a meta-analysis. PLoS One 8: e69366. 23894459 10.1371/journal.pone.0069366 PMC3716643

[16] Wolf S, Mertens D, Pscherer A, Schroeter P, Winkler D, et al (2006) Ala228 variant of trail receptor 1 affecting the ligand binding site is associated with chronic lymphocytic leukemia, mantle cell lymphoma, prostate cancer, head and neck squamous cell carcinoma and bladder cancer. Int J Cancer 118: 1831–1835. 16217763 10.1002/ijc.21502

[17] Sibley K, Rollinson S, Allan JM, Smith AG, Law GR, et al (2003) Functional FAS promoter polymorphisms are associated with increased risk of acute myeloid leukemia. Cancer Res 63: 4327–4330. 12907599

[18] Wu J, Metz C, Xu X, Abe R, Gibson AW, et al (2003) A novel polymorphic CAAT/enhancer-binding protein beta element in the FasL gene promoter alters Fas ligand expression: a candidate background gene in African American systemic lupus erythematosus patients. J Immunol 170: 132–138. 12496392 10.4049/jimmunol.170.1.132

[19] Mittal RD, Srivastava P, Mittal T, Verma A, Jaiswal PK, et al (2011) Association of death receptor 4, Caspase 3 and 5 gene polymorphism with increased risk to bladder cancer in North Indians. Eur J Surg Oncol 37: 727–733. 21700414 10.1016/j.ejso.2011.05.013

[20] Gormus U, Ergen A, Yilmaz H, Dalan B, Berkman S, et al (2007) Fas-1377A/G and FasL-844 T/C gene polymorphisms and epithelial ovarian cancer. Anticancer Res 27: 991–994. 17465232

[21] Horak P, Pils D, Roessler M, Tomek S, Elandt K, et al (2005) Common death receptor 4 (DR4) polymorphisms do not predispose to ovarian cancer. Gynecol Oncol 97: 514–518. 15863153 10.1016/j.ygyno.2005.01.021

[22] Frank B, Shanmugam KS, Beckmann L, Hemminki K, Brenner H, et al (2006) Death receptor 4 variants and colorectal cancer risk. Cancer Epidemiol Biomarkers Prev 15: 2002–2005. 17035413 10.1158/1055-9965.EPI-06-0053

[23] Hsu PI, Lu PJ, Wang EM, Ger LP, Lo GH, et al (2008) Polymorphisms of death pathway genes FAS and FASL and risk of premalignant gastric lesions. Anticancer Res 28: 97–103. 18383830

[24] Zhou RM, Wang N, Chen ZF, Duan YN, Sun DL, et al (2010) Polymorphisms in promoter region of FAS and FASL gene and risk of cardia gastric adenocarcinoma. J Gastroenterol Hepatol 25: 555–561. 20074157 10.1111/j.1440-1746.2009.06116.x

[25] Martinez-Ferrandis JI, Rodriguez-Lopez R, Milne RL, Gonzalez E, Cebolla E, et al (2007) Polymorphisms in TRAIL receptor genes and risk of breast cancer in Spanish women. Cancer Biomark 3: 89–93. 17522430 10.3233/cbm-2007-3203

[26] Hashemi M, Fazaeli A, Ghavami S, Eskandari-Nasab E, Arbabi F, et al (2013) Functional polymorphisms of FAS and FASL gene and risk of breast cancer - pilot study of 134 cases. PLoS One 8: e53075. 23326385 10.1371/journal.pone.0053075 PMC3543397

[27] Ter-Minassian M, Zhai R, Asomaning K, Su L, Zhou W, et al (2008) Apoptosis gene polymorphisms, age, smoking and the risk of non-small cell lung cancer. Carcinogenesis 29: 2147–2152. 18757527 10.1093/carcin/bgn205 PMC2577138

[28] Ulybina YM, Kuligina E, Mitiushkina NV, Rozanov ME, Ivantsov AO, et al (2009) Coding polymorphisms in Casp5, Casp8 and DR4 genes may play a role in predisposition to lung cancer. Cancer Lett 278: 183–191. 19203830 10.1016/j.canlet.2009.01.012

[29] Zhang Z, Wang LE, Sturgis EM, El-Naggar AK, Hong WK, et al (2006) Polymorphisms of FAS and FAS ligand genes involved in the death pathway and risk and progression of squamous cell carcinoma of the head and neck. Clin Cancer Res 12: 5596–5602. 17000697 10.1158/1078-0432.CCR-05-1739

[30] Shao P, Ding Q, Qin C, Wang M, Tang J, et al (2011) Functional polymorphisms in cell death pathway genes FAS and FAS ligand and risk of prostate cancer in a Chinese population. Prostate 71: 1122–1130. 21557277 10.1002/pros.21328

[31] Edge SB, Compton CC (2010) The American Joint Committee on Cancer: the 7th edition of the AJCC cancer staging manual and the future of TNM. Ann Surg Oncol 17: 1471–1474. 20180029 10.1245/s10434-010-0985-4

[32] Rai R, Sharma KL, Tiwari S, Misra S, Kumar A, et al (2013) DCC (deleted in colorectal carcinoma) gene variants confer increased susceptibility to gallbladder cancer (Ref. No.: Gene-D-12-01446). Gene 518: 303–309. 23353777 10.1016/j.gene.2013.01.019

[33] Miller SA, Dykes DD, Polesky HF (1988) A simple salting out procedure for extracting DNA from human nucleated cells. Nucleic Acids Res 16: 1215. 3344216 10.1093/nar/16.3.1215 PMC334765

[34] Gauderman W, Morrison J (2006) QUANTO 1.1: A computer program for power andsample size calculations for genetic-epidemiology studies. http://hydra.usc.edu/gxe.

[35] Sole X, Guino E, Valls J, Iniesta R, Moreno V (2006) SNPStats: a web tool for the analysis of association studies. Bioinformatics 22: 1928–1929. 16720584 10.1093/bioinformatics/btl268

[36] Carlson JM, Heckerman D, Shani G, (2009) Estimating False Discovery Rates for Contingency Tables. Microsoft research technical report MSR-TR-2009-53.37. http://research.microsoft.com/en-us/um/redmond/projects/MSCompBio/FalseDiscoveryRate/.

[37] Cochran W (1954) The combination of estimates from different experiments. Biometrics 10: 101–129.

[38] Egger M, Davey Smith G, Schneider M, Minder C (1997) Bias in meta-analysis detected by a simple, graphical test. BMJ 315: 629–634. 9310563 10.1136/bmj.315.7109.629 PMC2127453

[39] Thakkinstian A, McElduff P, D'Este C, Duffy D, Attia J (2005) A method for meta-analysis of molecular association studies. Stat Med 24: 1291–1306. 15568190 10.1002/sim.2010

[40] Tastemir-Korkmaz D, Demirhan O, Kuleci S, Hasturk S (2013) There is no Significant Association Between Death Receptor 4 (DR4) Gene Polymorphisms and Lung Cancer in Turkish Population. Pathol Oncol Res 19: 779–784. 23661154 10.1007/s12253-013-9643-z

[41] Korner C, Riesner K, Kramer B, Eisenhardt M, Glassner A, et al (2012) TRAIL receptor I (DR4) polymorphisms C626G and A683C are associated with an increased risk for hepatocellular carcinoma (HCC) in HCV-infected patients. BMC Cancer 12: 85. 22401174 10.1186/1471-2407-12-85 PMC3372437

[42] Ulybina YM, Kuligina ES, Mitiushkina NV, Sherina NY, Baholdin DV, et al (2011) Distribution of coding apoptotic gene polymorphisms in women with extreme phenotypes of breast cancer predisposition and tolerance. Tumori 97: 248–251. 21617726 10.1177/030089161109700222

[43] Kuraoka K, Matsumura S, Sanada Y, Nakachi K, Imai K, et al (2005) A single nucleotide polymorphism in the extracellular domain of TRAIL receptor DR4 at nucleotide 626 in gastric cancer patients in Japan. Oncol Rep 14: 465–470. 16012731

[44] Fernandez V, Jares P, Bea S, Salaverria I, Guino E, et al (2004) Frequent polymorphic changes but not mutations of TRAIL receptors DR4 and DR5 in mantle cell lymphoma and other B-cell lymphoid neoplasms. Haematologica 89: 1322–1331. 15531454

[45] MacPherson G, Healey CS, Teare MD, Balasubramanian SP, Reed MW, et al (2004) Association of a common variant of the CASP8 gene with reduced risk of breast cancer. J Natl Cancer Inst 96: 1866–1869. 15601643 10.1093/jnci/dji001

[46] Hazra A, Chamberlain RM, Grossman HB, Zhu Y, Spitz MR, et al (2003) Death receptor 4 and bladder cancer risk. Cancer Res 63: 1157–1159. 12649168

[47] Wajant H (2003) Death receptors. Essays Biochem 39: 53–71. 14585074 10.1042/bse0390053

[48] Shirley S, Morizot A, Micheau O (2011) Regulating TRAIL receptor-induced cell death at the membrane: a deadly discussion. Recent Pat Anticancer Drug Discov 6: 311–323. 21756247 10.2174/157489211796957757 PMC3204462

[49] George GP, Mandal RK, Kesarwani P, Sankhwar SN, Mandhani A, et al (2012) Polymorphisms and haplotypes in caspases 8 and 9 genes and risk for prostate cancer: a case-control study in cohort of North India. Urol Oncol 30: 781–789. 21396853 10.1016/j.urolonc.2010.08.027

[50] Guo Lu, Xia Bing, Sog Qi, Li Chun (2007) Association of DR4 gene polymorphism in Chinese patients with gastroduodenal diseases. Wuhan Daxue Xuebao (Yixue Ban) 28: 93–95.

[51] Chen B, Liu S, Wang XL, Xu W, Li Y, et al (2009) TRAIL-R1 polymorphisms and cancer susceptibility: an evidence-based meta-analysis. Eur J Cancer 45: 2598–2605. 19643596 10.1016/j.ejca.2009.06.023

[52] Park JB, Lee JK, Park EY, Riew KD (2008) Fas/FasL interaction of nucleus pulposus and cancer cells with the activation of caspases. Int Orthop 32: 835–840. 17589843 10.1007/s00264-007-0410-1 PMC2898961

[53] Zhang Z, Qiu L, Wang M, Tong N, Li J (2009) The FAS ligand promoter polymorphism, rs763110 (-844C>T), contributes to cancer susceptibility: evidence from 19 case-control studies. Eur J Hum Genet 17: 1294–1303. 19337311 10.1038/ejhg.2009.45 PMC2986648

[54] Xu LN, Zou SQ, Wang JM (2005) Action and mechanism of Fas and Fas ligand in immune escape of gallbladder carcinoma. World J Gastroenterol 11: 3719–3723. 15968727 10.3748/wjg.v11.i24.3719 PMC4316023

[55] Chatterjee K, Engelmark M, Gyllensten U, Dandara C, van der Merwe L, et al (2009) Fas and FasL gene polymorphisms are not associated with cervical cancer but differ among Black and Mixed-ancestry South Africans. BMC Res Notes 2: 238. 19941645 10.1186/1756-0500-2-238 PMC2787520

[56] Kim HJ, Jin XM, Kim HN, Lee IK, Park KS, et al (2010) Fas and FasL polymorphisms are not associated with acute myeloid leukemia risk in Koreans. DNA Cell Biol 29: 619–624. 20438363 10.1089/dna.2010.1032

[57] Gormus U, Ergen A, Yaylim-Eraltan I, Yilmaz H, Turna A, et al (2007) Fas-1377 A/G polymorphism in lung cancer. In Vivo 21: 663–666. 17708363

[58] Wang M, Wu D, Tan M, Gong W, Xue H, et al (2009) FAS and FAS ligand polymorphisms in the promoter regions and risk of gastric cancer in Southern China. Biochem Genet 47: 559–568. 19565204 10.1007/s10528-009-9264-0

[59] Zhang Z, Xue H, Gong W, Wang M, Yuan L, et al (2009) FAS promoter polymorphisms and cancer risk: a meta-analysis based on 34 case-control studies. Carcinogenesis 30: 487–493. 19168581 10.1093/carcin/bgp016

[60] Das SN, Khare P, Singh MK, Sharma SC (2011) Fas receptor (CD95) & Fas ligand (CD178) expression in patients with tobacco-related intraoral squamous cell carcinoma. Indian J Med Res 134: 54–60. 21808135 PMC3171918

[61] Pan G, O'Rourke K, Chinnaiyan AM, Gentz R, Ebner R, et al (1997) The receptor for the cytotoxic ligand TRAIL. Science 276: 111–113. 9082980 10.1126/science.276.5309.111

