# Supplementary figures and images for: Death Receptor (DR4) Haplotypes Are Associated with Increased Susceptibility of Gallbladder Carcinoma in North Indian Population
Source: PLoS One. 2014 Feb 28;9(2):e90264. doi: 10.1371/journal.pone.0090264 (PMC3938657; doi:10.1371/journal.pone.0090264)

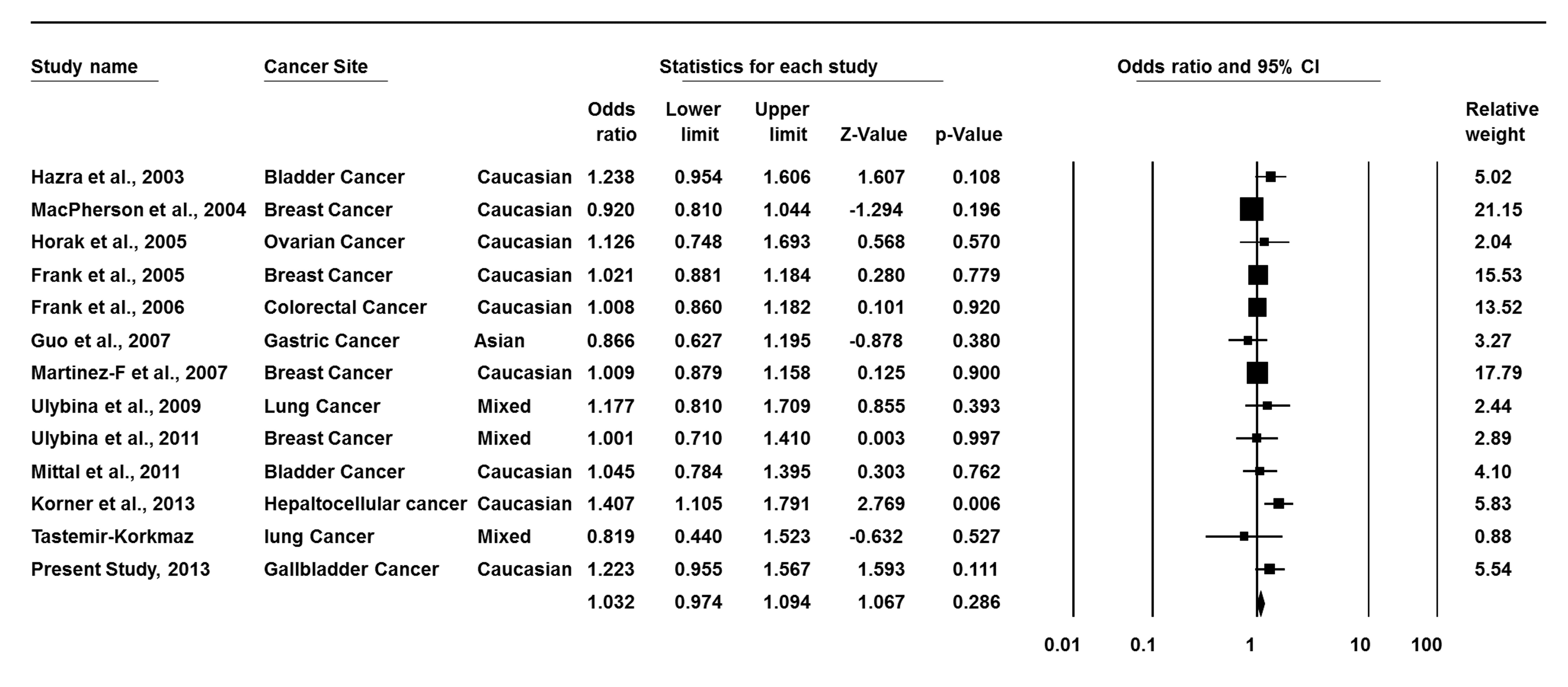

Supplement: File S1 — Figure S1. Forest plot for rs20575. Figure S2. Forest plot for rs205756. Figure S3. Forest plot for rs6557634. Figure S4. Funnel plot for rs20575. Each dot represents an individual study for the indicated association. The horizontal lines represent CIs. Figure S5. Funnel plot for rs205756. Each dot represents an individual study for the indicated association. The horizontal lines represent CIs. Figure S6. Funnel plot for rs6557634. Each dot represents an individual study for the indicated association. The horizontal lines represent CIs. Table S1. Frequency distribution of DR4, FAS and FASL Gene polymorphism in GBC and HC after subdividing on the basis of gender. Table S2. Frequency distribution of polymorphism in GBC and HC after subdividing on the basis of gallstone status. Table S3. Studies included in meta-analysis. Table S4: Functional information (ZIP) [file pone.0090264.s001.zip › si/Figure -s1a.tif]

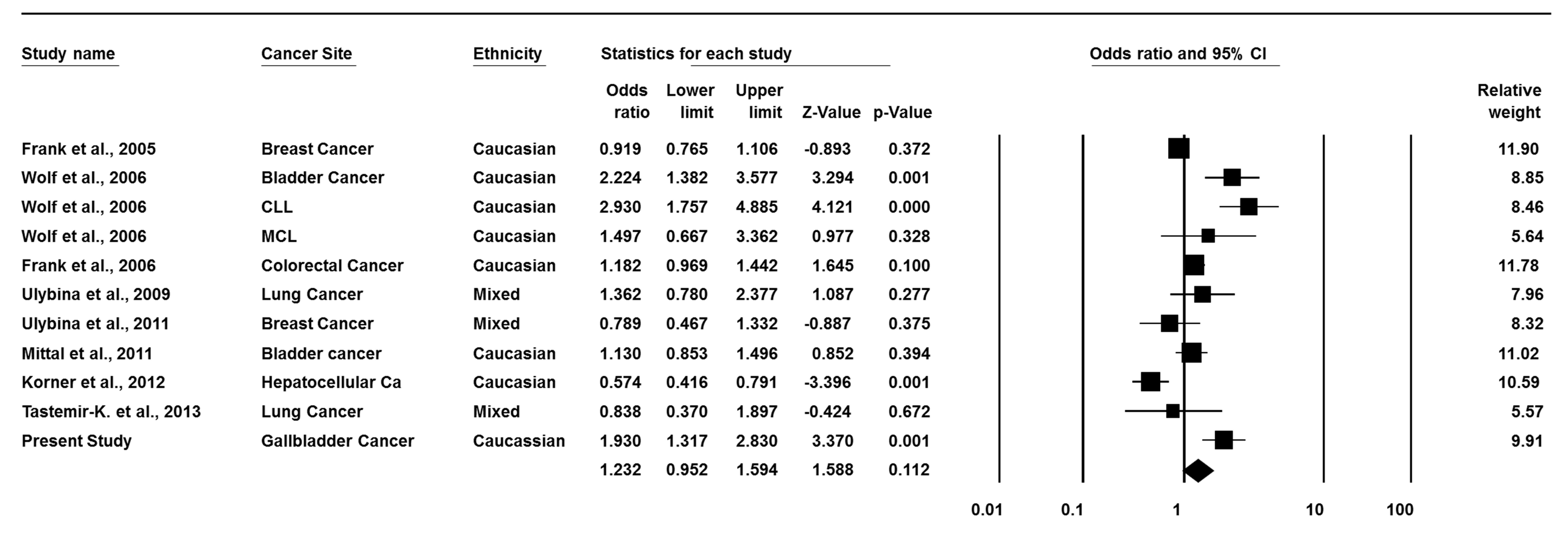

Supplement: File S1 — Figure S1. Forest plot for rs20575. Figure S2. Forest plot for rs205756. Figure S3. Forest plot for rs6557634. Figure S4. Funnel plot for rs20575. Each dot represents an individual study for the indicated association. The horizontal lines represent CIs. Figure S5. Funnel plot for rs205756. Each dot represents an individual study for the indicated association. The horizontal lines represent CIs. Figure S6. Funnel plot for rs6557634. Each dot represents an individual study for the indicated association. The horizontal lines represent CIs. Table S1. Frequency distribution of DR4, FAS and FASL Gene polymorphism in GBC and HC after subdividing on the basis of gender. Table S2. Frequency distribution of polymorphism in GBC and HC after subdividing on the basis of gallstone status. Table S3. Studies included in meta-analysis. Table S4: Functional information (ZIP) [file pone.0090264.s001.zip › si/Figure -s2a.tif]

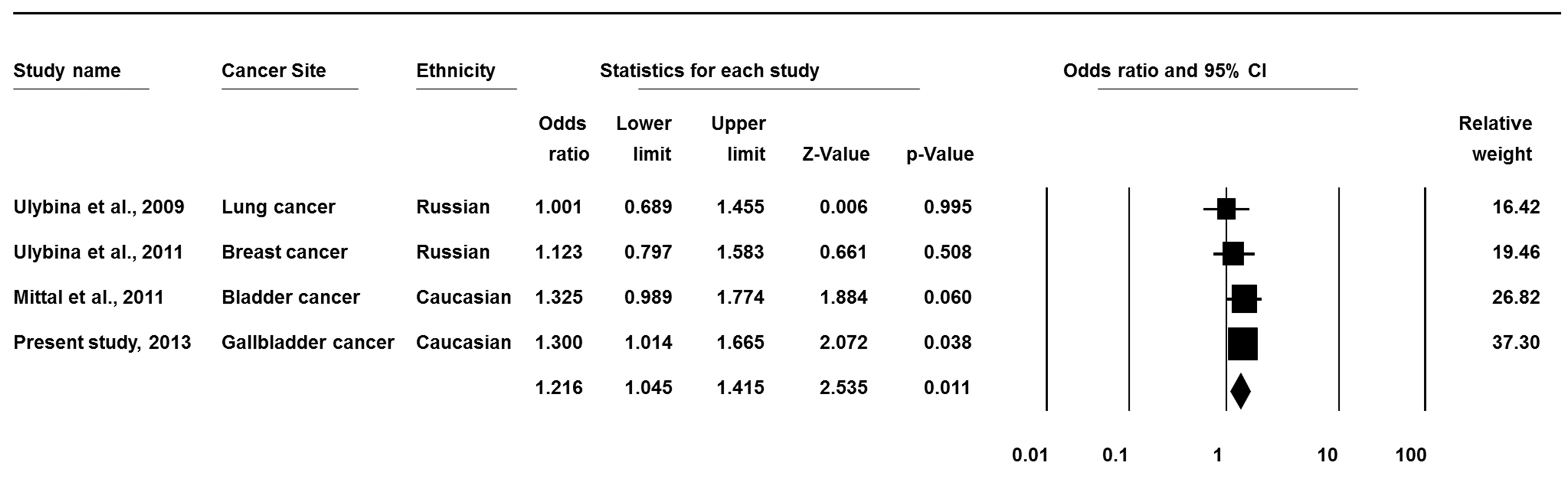

Supplement: File S1 — Figure S1. Forest plot for rs20575. Figure S2. Forest plot for rs205756. Figure S3. Forest plot for rs6557634. Figure S4. Funnel plot for rs20575. Each dot represents an individual study for the indicated association. The horizontal lines represent CIs. Figure S5. Funnel plot for rs205756. Each dot represents an individual study for the indicated association. The horizontal lines represent CIs. Figure S6. Funnel plot for rs6557634. Each dot represents an individual study for the indicated association. The horizontal lines represent CIs. Table S1. Frequency distribution of DR4, FAS and FASL Gene polymorphism in GBC and HC after subdividing on the basis of gender. Table S2. Frequency distribution of polymorphism in GBC and HC after subdividing on the basis of gallstone status. Table S3. Studies included in meta-analysis. Table S4: Functional information (ZIP) [file pone.0090264.s001.zip › si/Figure -s3a.tif]

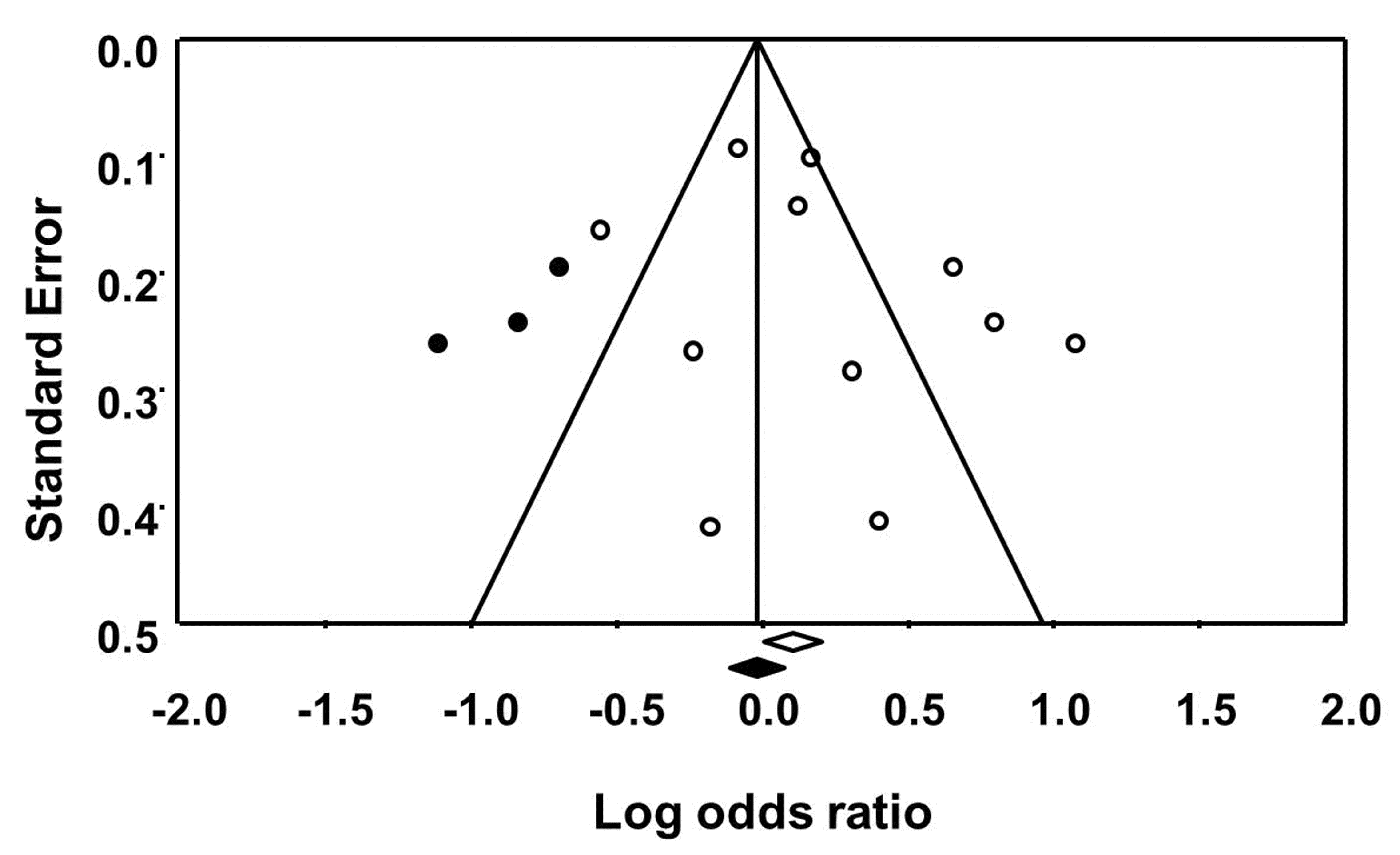

Supplement: File S1 — Figure S1. Forest plot for rs20575. Figure S2. Forest plot for rs205756. Figure S3. Forest plot for rs6557634. Figure S4. Funnel plot for rs20575. Each dot represents an individual study for the indicated association. The horizontal lines represent CIs. Figure S5. Funnel plot for rs205756. Each dot represents an individual study for the indicated association. The horizontal lines represent CIs. Figure S6. Funnel plot for rs6557634. Each dot represents an individual study for the indicated association. The horizontal lines represent CIs. Table S1. Frequency distribution of DR4, FAS and FASL Gene polymorphism in GBC and HC after subdividing on the basis of gender. Table S2. Frequency distribution of polymorphism in GBC and HC after subdividing on the basis of gallstone status. Table S3. Studies included in meta-analysis. Table S4: Functional information (ZIP) [file pone.0090264.s001.zip › si/Figure -s4a.tif]

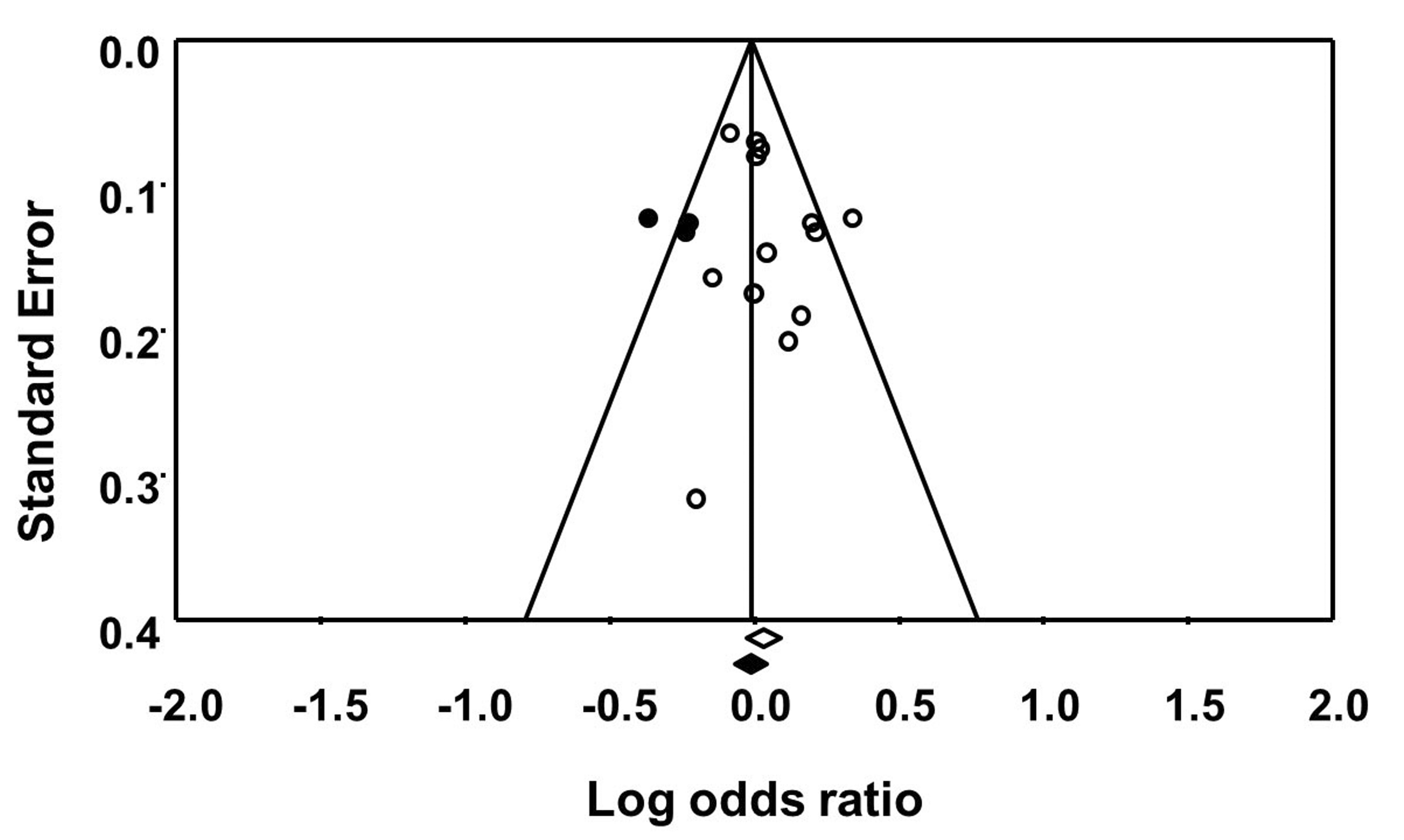

Supplement: File S1 — Figure S1. Forest plot for rs20575. Figure S2. Forest plot for rs205756. Figure S3. Forest plot for rs6557634. Figure S4. Funnel plot for rs20575. Each dot represents an individual study for the indicated association. The horizontal lines represent CIs. Figure S5. Funnel plot for rs205756. Each dot represents an individual study for the indicated association. The horizontal lines represent CIs. Figure S6. Funnel plot for rs6557634. Each dot represents an individual study for the indicated association. The horizontal lines represent CIs. Table S1. Frequency distribution of DR4, FAS and FASL Gene polymorphism in GBC and HC after subdividing on the basis of gender. Table S2. Frequency distribution of polymorphism in GBC and HC after subdividing on the basis of gallstone status. Table S3. Studies included in meta-analysis. Table S4: Functional information (ZIP) [file pone.0090264.s001.zip › si/Figure -s5a.tif]

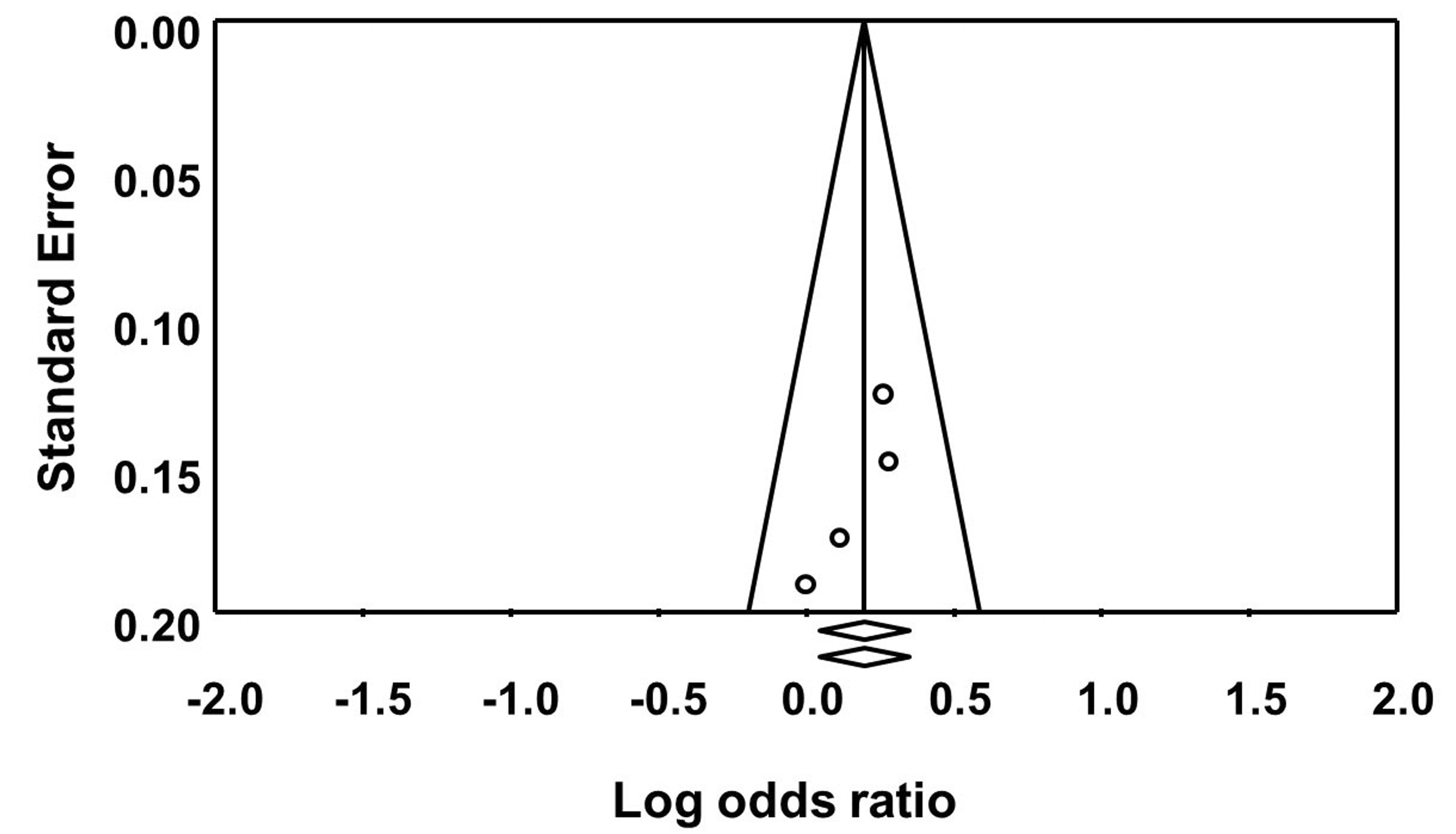

Supplement: File S1 — Figure S1. Forest plot for rs20575. Figure S2. Forest plot for rs205756. Figure S3. Forest plot for rs6557634. Figure S4. Funnel plot for rs20575. Each dot represents an individual study for the indicated association. The horizontal lines represent CIs. Figure S5. Funnel plot for rs205756. Each dot represents an individual study for the indicated association. The horizontal lines represent CIs. Figure S6. Funnel plot for rs6557634. Each dot represents an individual study for the indicated association. The horizontal lines represent CIs. Table S1. Frequency distribution of DR4, FAS and FASL Gene polymorphism in GBC and HC after subdividing on the basis of gender. Table S2. Frequency distribution of polymorphism in GBC and HC after subdividing on the basis of gallstone status. Table S3. Studies included in meta-analysis. Table S4: Functional information (ZIP) [file pone.0090264.s001.zip › si/Figure -s6a.tif]
