# Supplementary material for: Death Receptor (DR4) Haplotypes Are Associated with Increased Susceptibility of Gallbladder Carcinoma in North Indian Population
Source: PLoS One. 2014 Feb 28;9(2):e90264. doi: 10.1371/journal.pone.0090264 (PMC3938657; doi:10.1371/journal.pone.0090264)
Supplement: File S1 — Figure S1. Forest plot for rs20575. Figure S2. Forest plot for rs205756. Figure S3. Forest plot for rs6557634. Figure S4. Funnel plot for rs20575. Each dot represents an individual study for the indicated association. The horizontal lines represent CIs. Figure S5. Funnel plot for rs205756. Each dot represents an individual study for the indicated association. The horizontal lines represent CIs. Figure S6. Funnel plot for rs6557634. Each dot represents an individual study for the indicated association. The horizontal lines represent CIs. Table S1. Frequency distribution of DR4, FAS and FASL Gene polymorphism in GBC and HC after subdividing on the basis of gender. Table S2. Frequency distribution of polymorphism in GBC and HC after subdividing on the basis of gallstone status. Table S3. Studies included in meta-analysis. Table S4: Functional information (ZIP) [file pone.0090264.s001.zip › si/Supporting Information Table S1-s4 31dec2013.doc]

**Table S1. Frequency distribution of DR4, FAS and FASL Gene polymorphism in GBC and HC** after subdividing on the basis of gender

| **Genotype/**  **Allele** | **Male** | | | | **Female** | | | |
| --- | --- | --- | --- | --- | --- | --- | --- | --- |
|  | **Frequency N (%)** | | **p-value** | **OR (95%CI)** | **Frequency N (%)** | | **p-value** | **OR (95%CI)** |
|  | **HC (83)** | **GBC (122)** |  |  | **HC (163)** | **GBC (278)** |  |  |
| **DR4 rs20575 genotypes/alleles (age and gender adjusted)** | | | | | | | | |
| **GG** | 45 (54.2) | 63(51.6) | **-** | Reference | 83(50.9) | 120 (43.2) | - | Reference |
| **CG** | 31 (37.3) | 49 (40.2) | 0.632 | 0.83 (0.38-1.81) | 70 (42.9) | 132 (47.5) | 0.586 | 1.15 (0.70-1.88) |
| **CC** | 7 (8.4) | 10 (8.2) | 0.334 | 2.04 (0.48-8.67) | 10 (6.1) | 26 (9.4) | 0.138 | 1.98 (0.80-4.91) |
| **CG+CC** | 38 (45.8) | 59 (48.4) | 0.915 | 0.96 (0.46-2.01) | 80 (49.1) | 158 (56.8) | 0.354 | 1.25 (0.78-2.00) |
| **GG+CG vs. CC** | 76 (91.6) | 112 (91.8) | 0.275 | 2.20 (0.53-9.02) | 153 (93.9) | 252 (90.6) | 0.165 | 1.86 (0.78-4.44) |
| **G** | 121 (72.9) | 175 (71.7) | - | Reference | 236 (72.4) | 372 (66.9) | - | Reference |
| **C** | 45 (27.1) | 69 (28.3) | 0.710 | 1.12 (0.62-2.02) | 90 (27.6) | 184 (33.1) | 0.191 | 1.27 (0.89-1.83) |
| **DR4 rs20576** **genotypes/alleles (age and gender adjusted)** | | | | | | | | |
| **AA** | 73 (88.0) | 94 (77.0) | - | Reference | 137 (84.0) | 206 (74.1) | - | Reference |
| **AC** | 9 (10.8) | 23 (18.9) | 0.343 | 1.66 (0.58-4.73) | 24 (14.7) | 63 (22.7) | 0.070 | 1.76 (0.96-3.22) |
| **CC** | 1 (1.2) | 5 (4.1) | 0.633 | 2.27 (0.08-65.00) | 2 (1.2) | 9 (3.2) | 0.385 | 2.18 (0.38-12.61) |
| **AC+CC** | 10 (12.0) | 28 (23.0) | 0.303 | 1.70 (0.62-4.69) | 26 (16.0) | 72 (25.9) | 0.051 | 1.79 (1.00-3.21) |
| **AA+AC vs. CC** | 82 (98.8) | 117 (95.9) | 0.668 | 2.09 (0.07-61.31) | 161 (98.8) | 269 (96.8) | 0.458 | 1.94 (0.34-11.23) |
| **A** | 155 (93.4) | 211 (86.5) | - | Reference | 298 (91.4) | 475 (85.4) |  | Reference |
| **C** | 11 (6.6) | 33 (13.5) | 0.286 | 1.67 (0.65-4.25) | 28 (8.6) | 81 (14.6) | **0.046^a^** | **1.71 (1.01-2.90)** |
| **DR4 rs6557634 genotypes/alleles** **(age and gender adjusted)** | | | | | | | | |
| **GG** | 39 (47.0) | 51 (41.8) | - | Reference | 87 (53.4) | 123 (44.2) | - | Reference |
| **GA** | 40 (48.2) | 58 (47.5) | 0.201 | 1.66 (0.76-3.61) | 67 (41.1) | 134 (48.2) | 0.057 | 1.62 (0.99-2.65) |
| **AA** | 4 (4.8) | 13 (10.7) | 0.446 | 2.01 (0.33-12.06) | 9 (5.5) | 21 (7.6) | 0.137 | 2.01 (0.80-5.22) |
| **GA+AA** | 44 (53.0) | 71 (58.2) | 0.176 | 1.69 (0.79-3.61) | 76 (46.6) | 155 (55.8) | **0.034^b^** | **1.67 (1.04-2.69)** |
| **GG+GA vs. AA** | 79 (95.2) | 109 (89.3) | 0.629 | 1.53 (0.27-8.59) | 154 (94.5) | 257 (92.4) | 0.295 | 1.62 (0.65-4.04) |
| **G** | 118 (71.1) | 160 (65.6) | - | Reference | 241 (73.9) | 380 (68.3) | - | Reference |
| **A** | 48 (28.9) | 84 (34.4) | 0.236 | 1.41 (0.80-2.47) | 85 (26.1) | 176 (31.7) | **0.038^c^** | **1.49 (1.02-2.14)** |
| **FASL rs763110 genotypes/alleles** **(age and gender adjusted)** | | | | | | | | |
| **TT** | 36 (43.4) | 39 (32.0) | - | Reference | 54 (33.1) | 106 (38.1) | - | Reference |
| **CT** | 36 (43.4) | 64 (52.5) | 0.180 | 1.73 (0.78-3.85) | 83 (50.9) | 116 (41.7) | 0.279 | 0.75 (0.44-1.27) |
| **CC** | 11 (13.3) | 19 (15.6) | 0.984 | 1.01 (0.31-3.28) | 26 (16.0) | 56 (20.1) | 0.746 | 1.12 (0.57-2.20) |
| **CT+TT** | 47 (56.6) | 83 (68.0) | 0.265 | (1.53 (0.72-3.26) | 109 (66.9) | 172 (61.9) | 0.491 | 0.84 (0.52-1.37) |
| **TT+CT vs. CC** | 72 (86.7) | 103 (84.4) | 0.621 | 0.76 (0.25-2.27) | 137 (84.0) | 222 (79.9) | 0.384 | 1.31 (0.71-2.42) |
| **T** | 108 (65.1) | 142 (58.2) | - | Reference | 191 (58.6) | 328 (59.0) | - | Reference |
| **C** | 58 (34.9) | 102 (41.8) | 0.572 | 1.17 (0.68-2.01) | 135 (41.4) | 228 (41.0) | 0.990 | 1.00 (0.72-1.41) |
| **FAS rs2234767 genotypes/alleles** **(age and gender adjusted)** | | | | | | | | |
| **GG** | 43 (51.8) | 82 (67.2) | - | Reference | 110 (67.5) | 163 (58.6) | - | Reference |
| **AG** | 36 (43.4) | 36 (29.5) | 0.100 | 0.52 (0.24-1.24) | 50 (30.7) | 100 (36.0) | 0.394 | 1.25 (0.75-2.06) |
| **AA** | 4 (4.8) | 4 (3.3) | 0.095 | 0.14 (0.01-1.40) | 3 (1.8) | 15 (5.4) | 0.053 | 4.15 (0.98-17.58) |
| **AG+AA** | 40 (48.2) | 40 (32.8) | 0.056 | 0.47 (0.22-1.02) | 53 (32.5) | 115 (41.4) | 0.186 | 1.39 (0.85-2.26) |
| **GG+AG vs. AA** | 79 (95.2) | 118 (96.7) | 0.138 | 0.18 (0.02-1.74) | 160 (98.2) | 263 (94.6) | 0.065 | 3.85 (0.92-16.11) |
| **G** | 122 (73.5) | 200 (82.0) | - | Reference | 270 (82.8) | 426 (76.6) | - | Reference |
| **A** | 44 (26.5) | 44 (18.0) | **0.041^d^** | **0.51 (0.26-0.97)** | 56 (17.2) | 130 (23.4) | 0.079 | 1.45 (0.96-2.20) |

GBC- Gallbladder cancer, HC-Healthy Control, OR-Odds Ratio, CI-Confidence Interval. Significant Values are given in bold, FDR pcorr= 0.046^a^, 0.043^b^, 0.043^c^, 0.043^d^.

**Table S2. Frequency distribution of polymorphism in GBC and HC** after subdividing on the basis of gallstone status

| **Genotype/**  **Allele** | **HC (246)**  **N (%)** | **GBC without**  **stone (200)**  **N (%)** | **p –value** | **OR (95%CI)** | **GBC with stone (200)**  **N (%)** | **p–value** | **OR (95%CI)** |
| --- | --- | --- | --- | --- | --- | --- | --- |
| **DR4 rs20575 genotypes/alleles (age and gender adjusted)** | | | | | | | |
| **GG** | 128 (52.0) | 89 (44.5) | - | Reference | 94 (47.0) | - | Reference |
| **CG** | 101 (41.1) | 92 (46.0) | 0.492 | 1.18 (0.73-1.90) | 89 (44.5) | 0.813 | 9.38 (0.55-1.59) |
| **CC** | 17 (6.9) | 19 (9.5) | 0.176 | 1.81 (0.77-4.29) | 17 (8.5) | 0.116 | 2.18 (0.83 -5.77) |
| **CG+CC** | 118 (48.0) | 111 (55.5) | 0.312 | 1.27 (0.80-2.00) | 106 (53.0) | 0.801 | 1.07(0.65-1.76) |
| **GG+CG vs. CC** | 229 (93.1) | 181 (90.5) | 0.223 | 1.68 (0.73-3.85) | 183 (91.5) | 0.092 | 2.25 (0.88-5.76) |
| **G** | 357 (72.6) | 270 (67.5) | - | Reference | 277 (69.2) | - | Reference |
| **C** | 135 (27.4) | 130 (32.5) | 0.195 | 1.26 (0.89-1.79) | 123 (30.8) | 0.371 | 1.20 (0.81-1.77) |
| **DR4 rs20576** **genotypes/alleles (age and gender adjusted)** | | | | | | | |
| **AA** | 210 (85.4) | 149 (74.5) | - | Reference | 151 (75.5) | - | Reference |
| **AC** | 33 (13.4) | 45 (22.5) | 0.089 | 1.69 (0.92-3.08) | 41 (20.5) | **0.039^a^** | **1.97 (1.04-3.76)** |
| **CC** | 3 (1.2) | 6 (3.0) | 0.494 | 1.85 (0.32-10.86) | 8 (4.0) | 0.204 | 3.26 (0.53-20.17) |
| **AC+CC** | 36 (14.6) | 51 (25.5) | 0.072 | 1.70 (0.95-3.04) | 49 (24.5) | **0.021^b^** | **2.07 (1.12-3.85)** |
| **AA+AC vs. CC** | 243 (98.8) | 194 (97.0) | 0.563 | 1.69 (0.29-9.88) | 192 (96.0) | 0.260 | 2.84 (0.46-17.41) |
| **A** | 453 (92.1) | 343 (85.8) | - | Reference | 343 (85.8) | - | Reference |
| **C** | 39 (7.9) | 57 (14.2) | 0.070 | 1.63 (0.96-2.76) | 57 (14.2) | **0.014^c^** | **2.00 (1.15-3.48)** |
| **DR4 rs6557634 genotypes/alleles** **(age and gender adjusted)** | | | | | | | |
| **GG** | 126 (51.2) | 97 (48.5) | - | Reference | 77 (38.5) | - | Reference |
| **GA** | 107 (43.5) | 88 (44.0) | 0.438 | 1.21 (0.75-1.93) | 104 (52.0) | **0.000^d^** | **2.85 (1.63-4.99)** |
| **AA** | 13 (5.3) | 15 (7.5) | 0.745 | 0.85 (0.31-2.32) | 19 (9.5) | **0.004^e^** | **4.16 (1.59-10.88)** |
| **GA+AA** | 120 (48.8) | 103 (51.5) | 0.531 | 1.16 (0.73-1.83) | 123 (61.5) | **0.000^f^** | **3.03 (1.77-5.19)** |
| **GG+GA vs. AA** | 233 (94.7) | 185 (92.5) | 0.614 | 0.78 (0.29-2.08) | 181 (90.5) | 0.058 | 2.39 (0.97-5.88) |
| **G** | 359 (73.0) | 282 (70.5) | - | Reference | 258 (64.5) | - | Reference |
| **A** | 133 (27.0) | 118 (29.5) | 0.757 | 1.06 (0.74-1.52) | 142 (35.5) | **0.000^g^** | **2.15 (1.46-3.16)** |
| **FASL rs763110 genotypes/alleles** **(age and gender adjusted)** | | | | | | | |
| **TT** | 90 (36.6) | 82 (41.0) | - | Reference | 63 (31.5) | - | Reference |
| **CT** | 119 (48.4) | 85 (42.5) | 0.989 | 1.00 (0.61-1.65) | 95 (47.5) | 0.898 | 1.04 (0.59-1.81) |
| **CC** | 37 (15.0) | 33 (16.5) | 0.958 | 1.02 (0.52-2.00) | 42 (21.0) | 0.291 | 1.47 (0.72-3.02) |
| **CT+TT** | 156 (63.4) | 118 (59.0 | 0.976 | 1.01 (0.63-1.61) | 137 (68.5) | 0.610 | 1.15 (0.68-1.93) |
| **TT+CT vs. CC** | 209 (85.0) | 167 (83.5) | 0.959 | 1.02 (0.54-1.90) | 158 (79.0) | 0.267 | 1.44 (0.76-2.75) |
| **T** | 299 (60.8) | 249 (62.2) | - | Reference | 221 (55.2) | - | Reference |
| **C** | 193 (39.2) | 151 (37.8) | 0.961 | 1.01 (0.72-1.40) | 179 (44.8) | 0.333 | 1.19 (0.83-1.71) |
| **FAS rs2234767 genotypes/alleles** **(age and gender adjusted)** | | | | | | | |
| **GG** | 153 (62.2) | 124 (62.0) | - | Reference | 121 (60.5) | - | Reference |
| **AG** | 86 (35.0) | 66 (33.0) | 0.755 | 0.93 (0.57-1.50) | 70 (35.0) | 0.743 | 0.92 (0.54-1.55) |
| **AA** | 7 (2.8) | 10 (5.0) | 0.627 | 1.36 (0.39-4.69) | 9 (4.5) | 0.391 | 1.83 (0.46-7.30) |
| **AG+AA** | 93 (37.8) | 76 (38.0) | 0.863 | 0.96 (0.60-1.53) | 79 (39.5) | 0.920 | 0.97 (0.58-1.63) |
| **GG+AG vs. AA** | 239 (97.2) | 190 (95.0) | 0.593 | 1.40 (0.41-4.76) | 191 (95.5) | 0.362 | 1.89 (0.48-7.44) |
| **G** | 392 (79.7) | 314 (78.5) | - | Reference | 312 (78.0) | - | Reference |
| **A** | 100 (20.3) | 86 (21.5) | 0.979 | 1.01 (0.69-1.49) | 88 (22.0) | 0.839 | 1.05 (0.68-1.61) |

HC - Healthy Control, GBC-Gallbladder cancer, OR -Odds Ratio, CI-Confidence Interval

Significant Values are given in bold. FDR pcorr=0.043^a^, 0.034^b^, 0.028^c^, 0 ^d^, 0.014 ^e^, 0 ^f^, 0^g^.

**Table S3.** Studies included in meta-analysis

| **SN** | **Author** | **Ref.** | **Country** | **Ethnicity** | **Cancer type** | **Case/ Control** | **SNP** | **P_HWE_** | **MAF** | **Genotyping method** |
| --- | --- | --- | --- | --- | --- | --- | --- | --- | --- | --- |
| **1** | Tastemir-Korkmaz et al., 13 | 39 | Turkey | Mixed | Lung | 60/ 30 | rs20576  rs20575 | 0.4813  0.5605 | 18.33  46.67 | PCR-RFLP |
| **2** | Korner et al., 2012 | 40 | Germany | Caucasian | Hepatocellular Carcinoma | 215/359 | rs20576  rs20575 | 0.9888  0.9277 | 22.7  41.55 | Light Cycler RT-PCR |
| **3** | Mittal et al., 2011 | 17 | India | Caucasian | Bladder cancer | 200/ 225 | rs20576  rs20575  rs6557634 | 0.2433  0.0596  0.3498 | 34.44  31.78  27.78 | AS-PCR |
| **4** | Ulybina et al., 2011 | 41 | Russia | Mixed | Breast Cancer | 121/142 | rs20576  rs20575  rs6557634 | 0.4934  0.7816  0.5015 | 13.73  47.54  50.0 | Arms PCR |
| **5** | Ulybina et al., 2009 | 26 | Russia | Mixed | Lung Cancer | 111/110 | rs20576  rs20575  rs6557634 | 0.8602  0.5238  0.9437 | 11.36  48.64  46.82 | Arms PCR |
| **6** | Martinez-Ferrandis et al., 2007 | 23 | Spain | Caucasian | Breast cancer | 1008/768 | rs20575 | 0.6534 | 46.72 | Taqman |
| **7** | Guo-Lu et al., 2007 | 49 | China | Asian | Gastric cancer | 135/169 | rs20575 | 0.1782 | 46.15 | PCR-RFLP |
| **8** | Frank et al., 2006 | 20 | Germany | Caucasian | Colorectal cancer | 659/607 | rs20576  rs20575 | 0.8355  0.7749 | 18.61  45.8 | Taqman |
| **9** | Wolf et al., 2006 | 28 | Germany | Caucasian | CLL  MCL | 101/ 137 | rs20576  rs20576 | 0.4411  0.4411 | 9.85  9.85 | PCR |
|  |  |  |  |  |  | 32/ 137 |  |  |  |  |
| **10** | Frank et al., 2005 | 6 | Germany | Caucasian | Breast Cancer | 511/ 1092 | rs20576  rs20575 | 0.9186  0.4897 | 21.34  45.91 | Taqman |
|  |  |  |  |  |  | 519/ 1100 |  |  |  |  |
| **11** | Horak et al., 2005 | 19 | Austria | Caucasian | Ovarian Cancer | 92/ 100 | rs20575 | 0.8786 | 39.0 | PCR-RFLP |
| **12** | MacPherson et al., | 44 | UK | Caucasian | Breast cancer | 958/ 958 | rs20575 | 0.5727 | 48.7 | Taqman |
| **13** | Hazra et al., 2003 | 45 | Texas | Caucasian | Bladder Cancer | 253/ 215 | rs20575 | 0.9745 | 40.23 | PCR-RFLP |

**Table S4. Functional information**

| **Functional Category** | **Prediction Tool** | **Prediction Result** |
| --- | --- | --- |
| **rs20575  (FS score =0.330)** | | |
| Protein coding | Ensembl | Nonsyn |
|  | SIFT | Tolerated |
|  | Polyphen | Benign |
|  | SNP effect | Benign |
| Splicing regulation | ESE finder | **Changed** |
|  | ESR Search | **Changed** |
|  | PESX | **Changed** |
| Post translation | OGPET | **Exist** |
| **rs20576 (FS score=0)** | | |
| Protein coding | Ensemble | Nonsyn |
|  | SIFT | Tolerated |
|  | Polyphen | **Possible damaging** |
| **rs6557634 (FS score=0.284)** | | |
| Protein coding | Ensembl | Nonsynonymous |
|  | Polyphen | **Probably damaging** |
|  | SNP effect | Benign |
| Splicing regulation | ESE finder | **Changed** |
|  | ESR Search | **Changed** |
|  | PESX | Not changed |
| **rs763110 (FS score=0.434)** | | |
| Protein coding | Ensembl | **Frameshift-coding** |
| Transcriptional regulation | TF-Search | **Changed** |
|  | Ensembl-TR | **regulatory_region** |
| **rs2234767 (FS score 0)** | | |
| Protein coding | Ensembl | Nonsyn |
|  | SIFT | Tolerated |
|  | Polyphen | Benign |
| Transcriptional regulation | Golden Path | Not-exist |
